# DLK proteins modulate NOTCH signaling to influence a brown or white 3T3-L1 adipocyte fate

**DOI:** 10.1038/s41598-018-35252-3

**Published:** 2018-11-16

**Authors:** María-Luisa Nueda, María-Julia González-Gómez, María-Milagros Rodríguez-Cano, Eva-María Monsalve, María José M. Díaz-Guerra, Beatriz Sánchez-Solana, Jorge Laborda, Victoriano Baladrón

**Affiliations:** 1 Área de Bioquímica y Biología Molecular, Dpto. Química Inorgánica, Orgánica y Bioquímica, Facultad de Medicina de Albacete/CRIB/Unidad de Biomedicina, Universidad de Castilla-La Mancha/CSIC, C/Almansa 14, 02008 Albacete, Spain; 2 Área de Bioquímica y Biología Molecular, Dpto. Química Inorgánica y Bioquímica, Facultad de Farmacia/CRIB/Unidad de Biomedicina, Universidad de Castilla-La Mancha/CSIC. C/Almansa 14, 02008 Albacete, Spain; 3 Laboratory of Cellular Oncology, National Cancer Institute (NCI), National Institutes of Health (NIH), Bethesda, MD USA

## Abstract

The role of NOTCH signaling in adipogenesis is highly controversial, with data indicating null, positive or negative effects on this differentiation process. We hypothesize that these contradictory results could be due to the different global NOTCH signaling levels obtained in different experimental settings, because of a specific modulation of NOTCH receptors’ activity by their ligands. We have previously demonstrated that DLK1 and DLK2, two non-canonical NOTCH1 ligands that inhibit NOTCH1 signaling in a dose-dependent manner, modulate the adipogenesis process of 3T3-L1 preadipocytes. In this work, we show that over-expression of any of the four NOTCH receptors enhanced adipogenesis of 3T3-L1 preadipocytes. We also determine that DLK proteins inhibit not only the activity of NOTCH1, but also the activity of NOTCH2, 3 and 4 receptors to different degrees. Interestingly, we have observed, by different approaches, that NOTCH1 over-expression seems to stimulate the differentiation of 3T3-L1 cells towards a brown-like adipocyte phenotype, whereas cells over-expressing NOTCH2, 3 or 4 receptors or DLK proteins would rather differentiate towards a white-like adipocyte phenotype. Finally, our data also demonstrate a complex feed-back mechanism involving *Notch* and *Dlk* genes in the regulation of their expression, which suggest that a precise level of global NOTCH expression and NOTCH-dependent transcriptional activity of specific targets could be necessary to determine the final phenotype of 3T3-L1 adipocytes.

## Introduction

NOTCH signaling is a key molecular pathway involved in several biological processes, including adipogenesis. It participates in a complex network of several signaling pathways that modulate the conversion of preadipocytes or mesenchymal stem cells into mature adipocytes. However, the role of NOTCH signaling in adipogenesis is highly controversial, and while some authors have claimed it to be dispensable for adipocyte differentiation^1^, others have shown positive or negative roles for NOTCH signaling in the adipogenesis process^2–7^. In addition, other works have reported a role for NOTCH signaling in energy metabolism and adipocyte browning^8–11^. The modulation of NOTCH signaling aimed at inhibiting the generation of white fat cells and stimulating the generation of brown adipocytes may constitute a promising strategy to regulate fat mass in humans.

Mammals possess four NOTCH receptors and five canonical ligands (JAGGED1 and 2, and DLL1, 3 and 4). The activation of NOTCH receptors is achieved by the interaction of the DSL (Delta-Serrate-Lag-2) N-terminal domain of a canonical ligand with specific extracellular EGF repeats of NOTCH receptors. Additional proteolytic events release the intracellular active domain of NOTCH receptors (NICD), which is relocated to the nucleus, and binds to a protein called CSL/RBP-Jk/CBF1 and to other co-activators to modulate the expression of several transcription factors, including those belonging to the HES/HEY family^12–15^.

The NOTCH protein family also includes some non-canonical ligands that inhibit NOTCH signaling, such as DLK1(Delta-like 1 homolog) and DLK2 (Delta-like 2 homolog)^16,17^, EGFL7 (Epidermal growth factor-like protein 7)^18^, or DNER (Delta/Notch Like EGF Repeat Containing)^19^. DLK1 and DLK2 are two homologous transmembrane proteins with six extracellular EGF-like repeats that interact with the NOTCH1 receptor and function as NOTCH signaling inhibitors^20–22^. DLK1 and DLK2 lack a DSL domain, although both possess a DOS domain that is postulated to function by competing with the canonical NOTCH receptor ligands^23^.

Accumulated evidence indicates that *Dlk1* is involved in several cell differentiation processes, including adipogenesis. Thus, several works, some of them performed in *Dlk1-*deficient and transgenic mice, point to *Dlk1* as an inhibitor of adipogenesis^22,24–28^. Several research groups, including ours, have demonstrated that DLK1 over-expression inhibits 3T3-L1 adipogenesis, whereas enforced decrease in *Dlk1* expression enhanced this differentiation process. Our research group furthermore demonstrated that *Dlk2* also modulates adipogenesis of 3T3-L1 cells^17^. On the other hand, recent studies implicate *Dlk1* in the control of whole body metabolism^29,30^, the onset of diabetes in humans^31,32^, and adipocyte browning^33,34^.

Several mechanisms have been proposed to explain the action of DLK proteins on 3T3-L1 adipogenesis^35–41^. However, probably the most important fact revealed by our and other research groups is that both proteins interact with the NOTCH1 receptor and function as inhibitory non-canonical ligands of NOTCH1 signaling in a dose-dependent manner^7,21,35–43^. We then hypothesized that these proteins could regulate adipogenesis and adipocyte phenotype by generating defined levels of NOTCH1 signaling, leading or not to the progression of this differentiation process. However, in our view, to achieve this precise level of NOTCH signaling, DLK proteins should not only modulate NOTCH1 activity, but also the activity of the other three NOTCH receptors.

In this work, we show that over-expression of any of the four NOTCH receptors enhanced the adipogenic potential of 3T3-L1 preadipocytes, and that DLK proteins can inhibit the activity of NOTCH1, 2, 3 and 4 receptors to different degrees. Interestingly, we have demonstrated by performing different assays that NOTCH1 over-expression drives differentiation of 3T3-L1 cells towards a brown-like adipocyte phenotype, whereas preadipocytes over-expressing NOTCH2, 3 or 4 receptors or DLK proteins develop a gene expression profile and phenotype more similar to white adipocytes. Finally, we also observed that over-expression of any of the four NOTCH receptors and their signaling and the over-expression of any DLK protein affect the expression levels of the others to different degrees. To sum up, all these data suggest the existence of a complex feedback regulation mechanism involving the expression of all *Notch* and *Dlk* genes that may lead to a precise level of NOTCH signaling to allow 3T3-L1 preadipocytes to differentiate or not to a particular adipocyte phenotype.

## Results

### Over-expression of each one of the NOTCH receptors enhances adipogenesis of 3T3-L1 preadipocytes

The role of NOTCH signaling in the adipogenesis process is a highly controversial topic. Rather than attributing the contradictory results to technical problems or cell line differences, we hypothesized that they could be related to the possibility that adipogenesis in response to extracellular signals may proceed or not given a precise level of global NOTCH receptor activation^22^.

**Figure 1:**
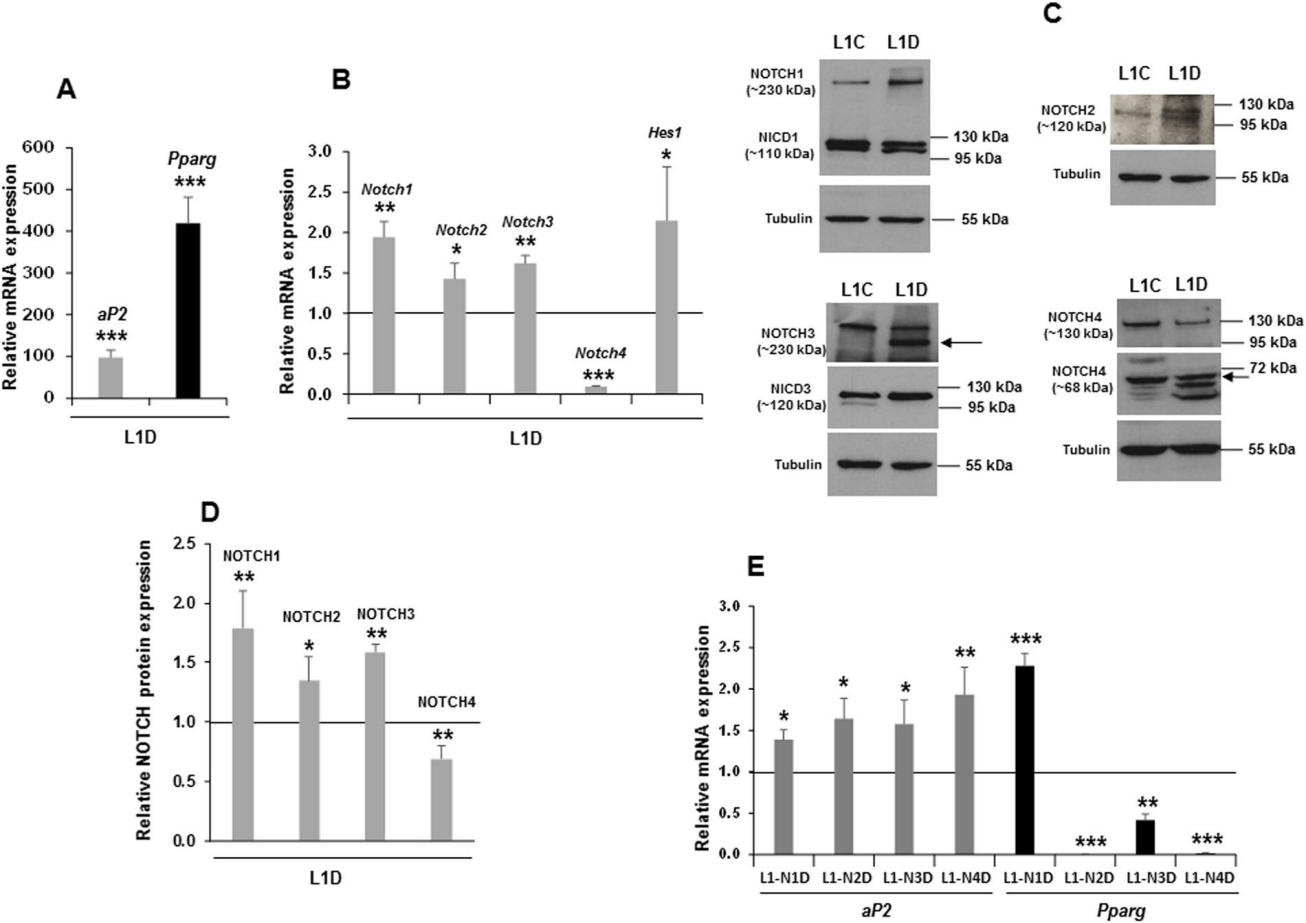
The stable over-expression of each one of the *Notch* genes on 3T3-L1 cells enhances adipogenesis. (**A**) qRT-PCR analysis of the relative mRNA expression levels of the adipocyte markers *aP2* and *Pparg* in differentiated 3T3-L1 cells. (**B**) qRT-PCR analysis of the relative *Notch* and *Hes1* mRNA expression levels in differentiated 3T3-L1 cells. Representative Western blots (**C**) and densitometric analysis (**D**) of each NOTCH receptor expression in 3T3-L1 adipocytes compared to non-differentiated cells. In the case of the *Notch1* gene transfectant intracellular NOTCH1 (NICD1) and complete NOTCH1 protein signals are shown. In the case of the *Notch2* gene transfectant, the intracellular NOTCH2 (NICD2) protein signal is shown. For the *Notch3* gene transfectant, the complete and the intracellular NOTCH3 (NICD3) protein signals are shown. Finally, for the *Notch*4 gene transfectant, the complete and the intracellular NOTCH4 (NICD4) protein signals are shown. (**E**) qRT-PCR analysis of the relative *aP2* and *Pparg* mRNA expression levels in differentiated stable *Notch1* gene transfectant (L1-N1D), stable *Notch2* gene transfectant (L1-N2D), stable *Notch3* gene transfectant (L1-N3D), and stable gene *Notch4* transfectant (L1-N4D). Data from qRT-PCR assays were previously normalized to *P0* mRNA expression levels. The expression of alpha-tubulin was used as a loading control in all Western blots to normalize expression data. Blot signals from empty vector and over-expressing cells were cropped from original blots and delineated with horizontal white spaces (original blots for each protein signal are shown in Supplementary Figure 4). The fold activation or inhibition was calculated relative to the seven-day differentiated non-transfected or empty-vector-transfected cells, which was set arbitrarily at 1. Data are shown as the mean ± SD of at least three biological assays performed in triplicate. The statistical significance calculated by Student’s t-tests is indicated (*p ≤ 0.05, **p ≤ 0.01, ***p ≤ 0.001).

We first analyzed here the basal mRNA expression levels of *Notch* genes in 3T3-L1 cells and found that these cells express similar levels of the four *Notch* genes (Supplementary Figure 1A). We observed that the induction of adipogenesis in 3T3-L1 cells increased *aP2 and Pparg* mRNA expression (Fig. 1A). In addition, whereas the expression of *Notch1*, *Notch2*, *Notch3*, and *Hes1* mRNAs increased with the adipogenic treatment, the expression of *Notch4* mRNA decreased (Fig. 1B). Western blot analysis also demonstrated that NOTCH4 protein expression decrease at the end of the adipogenic process, whereas the expression of NOTCH1, 2 and 3 increases (Fig. 1C,D).

Next, pools of 3T3-L1 preadipocytes stably over-expressing each one of the *Notch* genes were used to investigate the effects of each NOTCH receptor on 3T3-L1 adipogenesis. The over-expression of *Notch* genes and their proteins was confirmed by qRT-PCR and Western blot analysis (Supplementary Figure 1(B–I)). We have observed that over-expression of any *Notch* gene enhanced the adipogenic potential of these cells, as indicated by an increase in *aP2* expression (Fig. 1E). *Pparg* expression also increased in cells over-expressing *Notch1* gene, but its levels drastically decreased in differentiated cells that over-expressed *Notch2*, *Notch3* and *Notch4* genes (Fig. 1E).

### NOTCH receptors and DLK proteins modulate 3T3-L1 adipocyte browning

**Figure 2:**
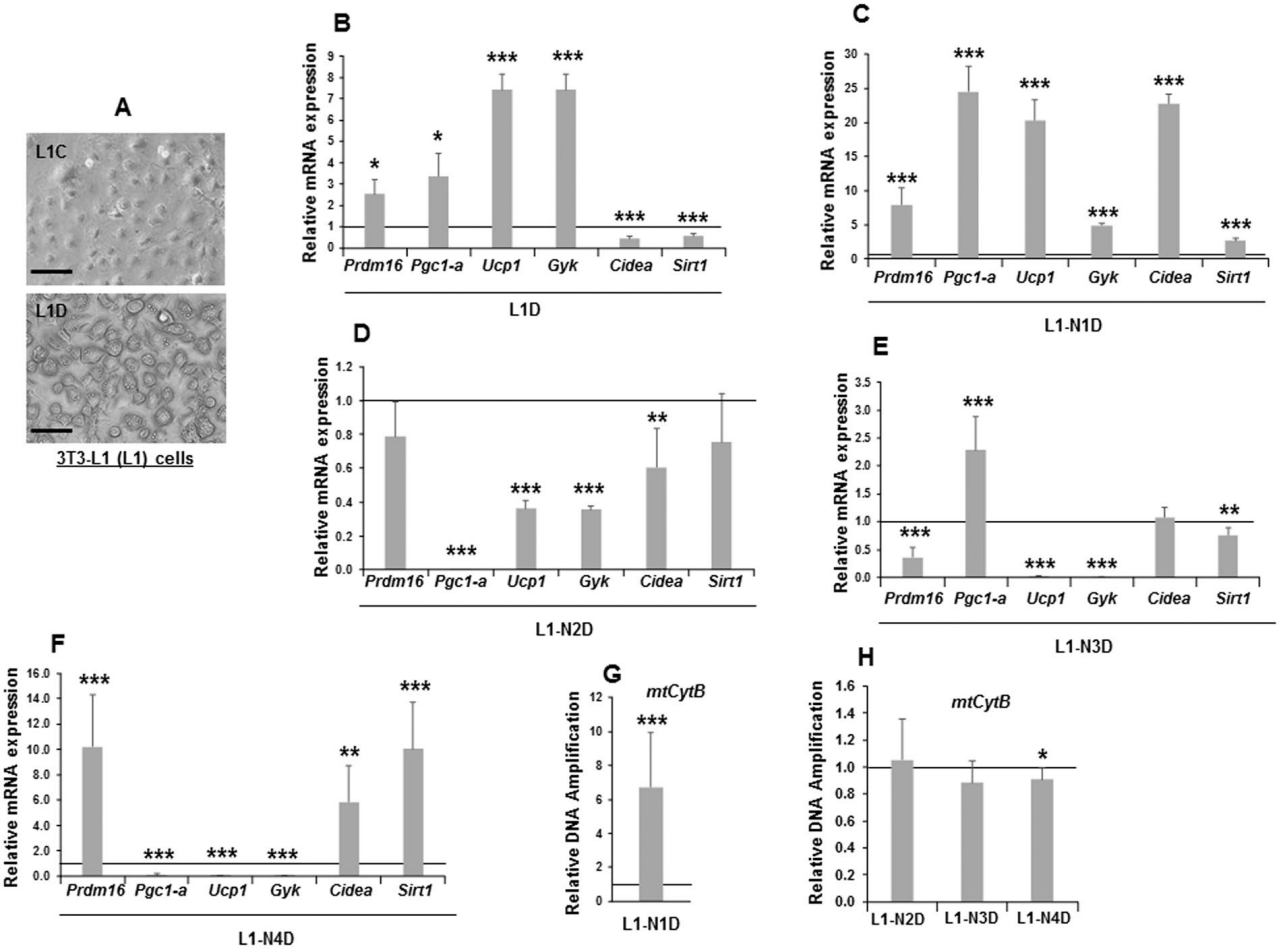
Effects of stable over-expression of each one of the *Notch* genes in 3T3-L1 adipocyte browning. (**A**) Representative microscopy images (400X magnification) of 3T3-L1 adipocytes (L1D) seven days after standard adipogenic induction (48 hours with IBMX and dexamethasone, and 5 days with insulin, see Methods) and non-treated 3T3-L1 cells (L1C). Scale bar (250 μm) is shown. (**B**) qRT-PCR mRNA expression analysis of the brown adipocyte markers *Ucp1*, *Pgc1a*, *Gyk*, *Prdm16*, *Cidea and Sirt1* in seven-day-differentiated 3T3-L1 cells. qRT-PCR analysis of the relative mRNA expression levels of *Ucp1*, *Pgc1a*, *Gyk*, *Prdm16*, *Cidea and Sirt1* markers in seven-day differentiated *Notch1* gene transfectant (L1-N1D) (**C**), *Notch2* gene transfectant (L1-N2D) (**D**), *Notch3* gene transfectant (L1-N3D) (**E**), *Notch4* gene transfectant (L1-N4D) (**F**). Data from qRT-PCR assays were previously normalized to *P0* mRNA expression levels. qPCR analysis of mitochondrial *CytB* DNA amplification (related to genomic ApoB DNA amplification, see Methods) in seven-day differentiated 3T3-L1 cells over-expressing *Notch1* gene (**G**) and *Notch2*, *Notch3* or *Notch4* genes (**H**). The fold activation or inhibition was calculated relative to the seven-day differentiated non-transfected or empty-vector-transfected cells, which was set arbitrarily at 1. Data are shown as the mean ± SD of at least three biological assays performed in triplicate. The statistical significance of the Student’s t-tests performed is indicated (*p ≤ 0.05, **p ≤ 0.01, ***p ≤ 0.001).

3T3-L1 preadipocytes have been always referred to as a typical cellular model of white adipogenesis. However, some authors described that 3T3-L1 adipocytes display phenotypic characteristics of multiple adipocyte lineages^44^. In this work, we have observed that the induction of adipogenesis in 3T3-L1 cells generates adipocytes (L1D) with multilocular lipid droplets (Fig. 2A), and increases the expression of the brown adipocyte and mitochondrial biogenesis markers *Pgc1a*, *Ucp1*, *Gyk* and *Prdm16* (Fig. 2B). We also observed a decrease in *Cidea* expression, which might be associated with the multilocular droplets observed^45^. However, the expression of the mitochondrial biogenesis marker *Sirt1* decreased in 3T3-L1 differentiated cells (Fig. 2B).

Taking these results into consideration, we were interested in exploring whether the stable over-expression of each one of the four NOTCH receptors could also affect the expression levels of the brown adipocyte genes after induction of adipogenesis (Fig. 2). Compared with differentiated control cells, *Notch1* over-expressing adipocytes showed higher expression levels of the brown adipocyte and the mitochondrial biogenesis markers *Pgc1a*, *Ucp1*, *Gyk*, *Prdm16*, *Cidea and Sirt1* (Fig. 2C). Likewise, *Notch2* over-expression diminished *Pgc1a*, *Ucp1*, *Gyk*, and *Cidea* expression, although no significant differences were observed in *Prdm16* and *Sirt1* expression (Fig. 2D). *Notch3* over-expression diminished the expression of *Ucp1*, *Gyk*, *Prdm16* and *Sirt1* in differentiated cells, although *Pgc1a* levels were increased and no significant differences were observed in *Cidea* expression (Fig. 2E). Finally, *Notch4* over-expression diminished the expression levels of *Pgc1a*, *Ucp and Gyk* in differentiated cells, but it increased the expression of *Prdm16*, *Cidea* and *Sirt1* (Fig. 2F). We also analyzed the amplification levels of mitochondrial *CytB* gene in these differentiated stable transfectants (see Methods), which indicates the rate of mitochondrial biogenesis. The over-expression of *Notch1* increases the amplification level of mitochondrial *CytB* gene in differentiated cells (Fig. 2G), whereas the over-expression of *Notch4* decreases the amplification of this gene (Fig. 2H). No significant differences in mitochondrial *CytB* gene amplification were found when cells over-express *Notch2* or *Notch3* genes (Fig. 2H).

**Figure 3:**
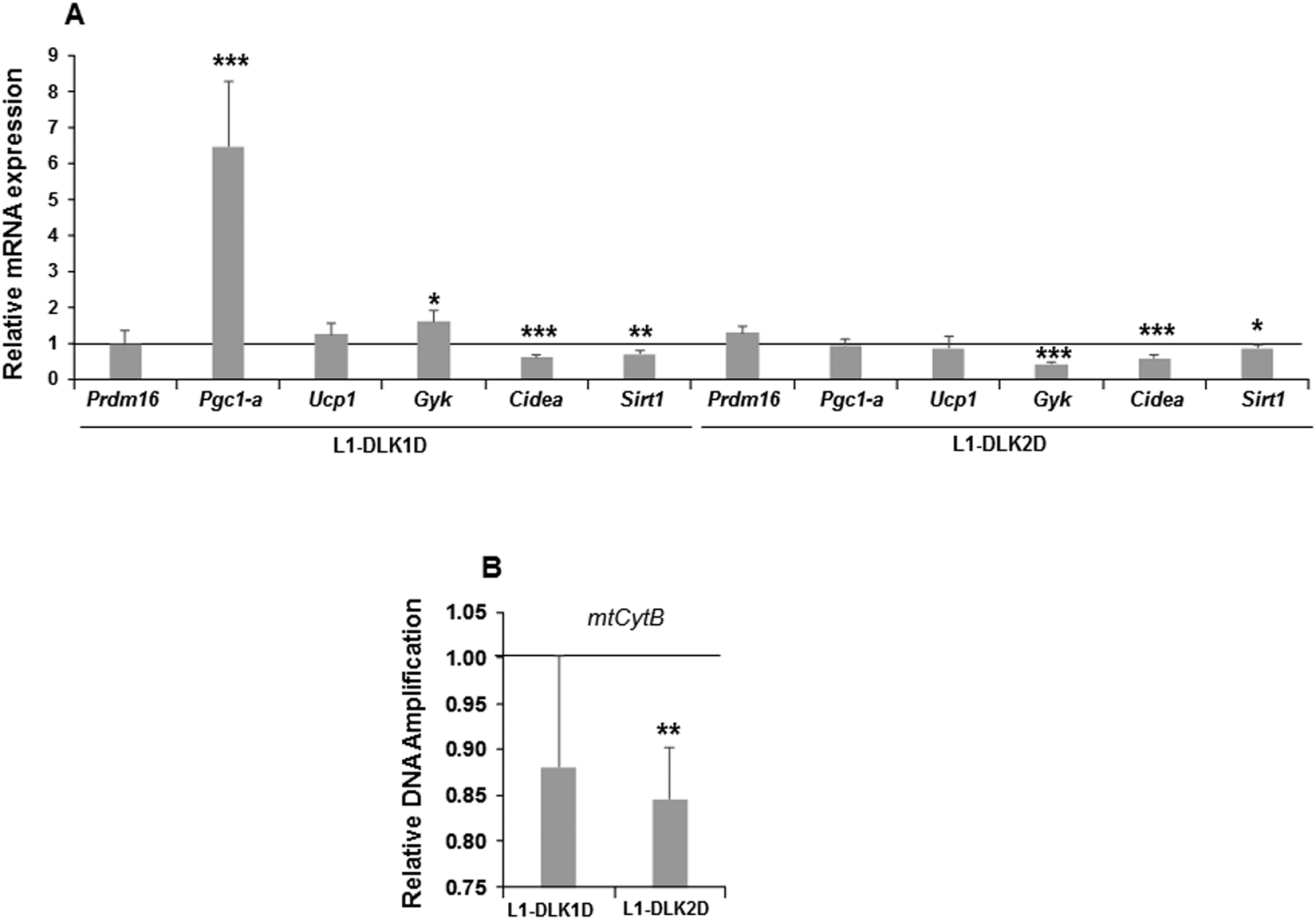
Effects of stable over-expression of *Dlk1* or *Dlk2* genes in 3T3-L1 adipocyte browning. (**A**) qRT-PCR analysis of the relative mRNA expression levels of *Ucp1*, *Pgc1a*, *Gyk*, *Prdm16*, *Cidea and Sirt1* markers in seven-day differentiated *Dlk1* gene transfectant (L1-DLK1D) and *Dlk2* gene transfectant (L1-DLK2D). Data from qRT-PCR assays were previously normalized to *P0* mRNA expression levels. (**B**) qPCR analysis of mitochondrial *CytB* DNA amplification (related to genomic ApoB DNA amplification, see Methods) in seven-day differentiated 3T3-L1 cells over-expressing *Dlk1* or *Dlk2* genes. The fold activation or inhibition was calculated relative to the seven-day differentiated non-transfected or empty-vector-transfected cells, which was set arbitrarily at 1. Data are shown as the mean ± SD of at least three biological assays performed in triplicate. The statistical significance of the Student’s t-tests performed is indicated (*p ≤ 0.05, **p ≤ 0.01, ***p ≤ 0.001).

Being inhibitors of NOTCH signaling, we expected that stable over-expression of *Dlk1* or *Dlk2* would affect also the expression of these markers (Fig. 3). Thus, we generated 3T3-L1 cells over-expressing *Dlk1* or *Dlk2* (Supplementary Figure 2) to study this hypothesis. We observed that *Dlk1* over-expression significantly increased the expression of *Pgc1a* and *Gyk*, decreased that of *Cidea* and *Sirt1*, and left that of *Ucp1* and *Prdm16* unaffected (Fig. 3A). On the other hand, cells stably over-expressing *Dlk2* showed a decrease in *Gyk*, *Cidea*, *Sirt1* expression, although no significant differences were observed in *Pgc1a*, *Prdm16* and *Ucp1* expression (Fig. 3A). We also analyzed the amplification levels of mitochondrial *CytB* gene amplification in these stable transfectants (see Methods). The over-expression of *Dlk2* decreases the amplification levels of this gene (Fig. 3B). No significant differences in mitochondrial *CytB* gene amplification were found when cells over-express *Dlk1* gene (Fig. 3B).

**Figure 4:**
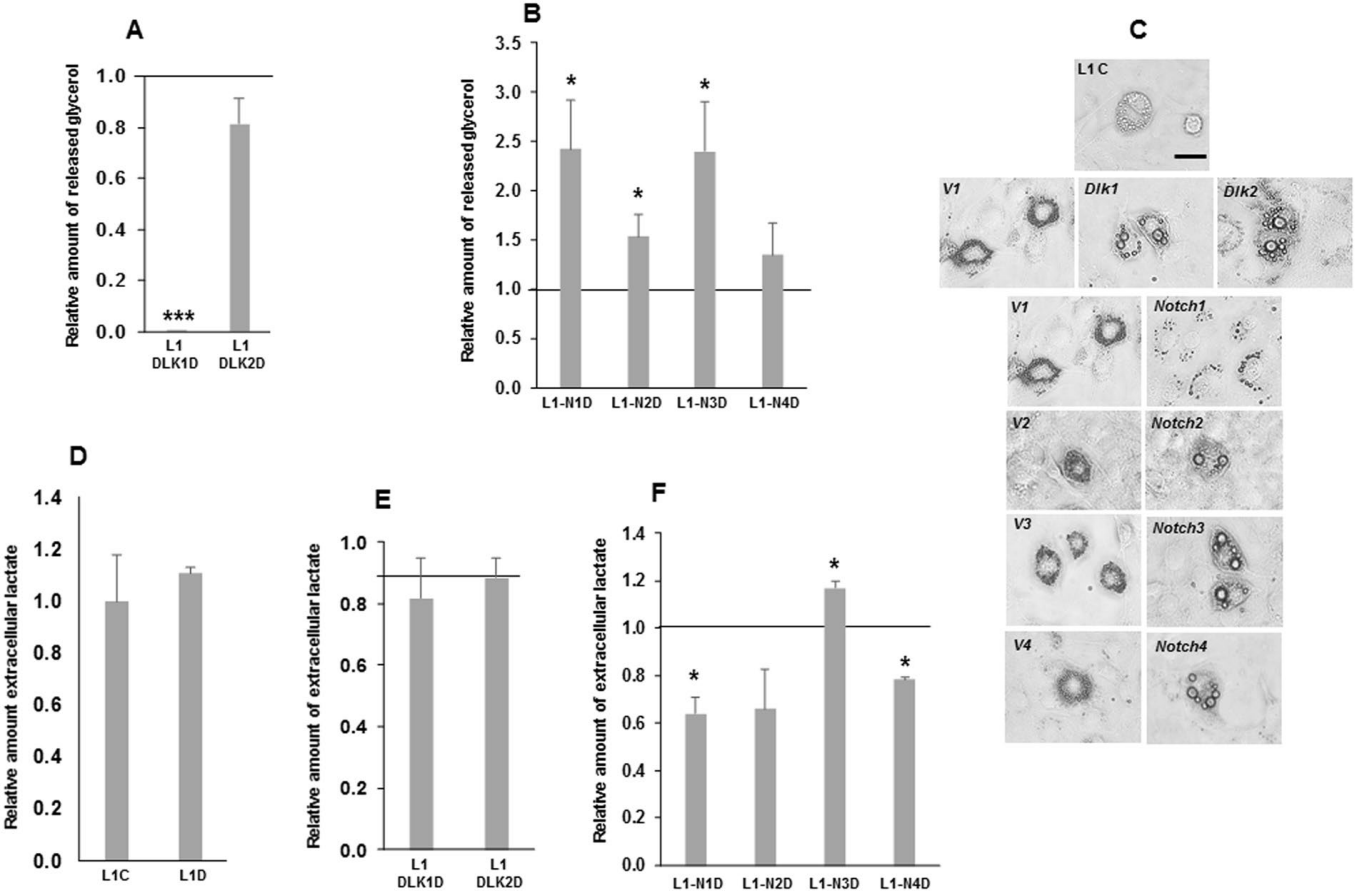
Release of glycerol and lactate to the extracellular medium in 3T3-L1 adipocytes over-expressing *Dlk* or *Notch* genes. Relative levels of glycerol released to the extracellular medium in response to isoproterenol from *Dlk1* or *Dlk2* genes (**A**), and *Notch1*, *2*, *3* or *4* genes **(B)** over-expressing  adipocytes. (**C**) Representative microscopy images (400X magnification) of non-transfected (L1C) and transfected 3T3-L1 adipocytes (Empty vectors V1, V2, V3 and V4, and their corresponding over-expressing transfectant) under study. The size of their lipid droplets is showed. Scale bar (80 μm) is shown. Relative levels of lactate in the culture supernatant of differentiated non-transfected 3T3-L1 cells (**D**), *Dlk1* or *Dlk2* genes over-expressing  adipocytes (**E**), and each of the *Notch* genes over-expressing  adipocytes (**F**). The fold activation or inhibition was calculated relative to the seven-day differentiated non-transfected or empty-vector-transfected cells, which was set arbitrarily at 1. Data are shown as the mean ± SD of at least three biological assays performed in triplicate. The statistical significance calculated by Student’s t-tests is indicated (*p ≤ 0.05, **p ≤ 0.01, ***p ≤ 0.001).

To estimate the cytoplasmic lipid accumulation, we measured the amount of glycerol released from the different transfected adipocytes by inducing lipolysis with the β-adrenergic agonist isoproterenol (Fig. 4). 3T3-L1 transfectants over-expressing *Dlk1* released lower glycerol levels into the medium than control cells (Fig. 4A), whereas the transfectants over-expressing *Notch1*, *Notch2* or *Notch3* showed higher levels of glycerol release when compared with their respective control cells (Fig. 4B). No significant differences were observed in cells over-expressing *Dlk2* or *Notch4* (Fig. 4A,B). We also observed that 80–90% of 3T3-L1 adipocytes over-expressing *Dlk1*, *Dlk2*, *Notch2*, *Notch3* or *Notch4* genes contained larger lipid droplets than their corresponding controls or non-transfected 3T3-L1 differentiated cells (L1C) (Fig. 4C). However, differentiated *Notch1* over-expressing cells, which show an increase in the number and size of adipocytes compared with their controls, exhibited a reduction in the multilocular lipid droplet size (Fig. 4C).

To further characterize at a functional level the different types of adipocytes derived from each transfectant, we also analysed the lactate released into the extracellular medium, which indirectly provides the extra-cellular acidification rate (ECAR) (Fig. 4). Compared to their corresponding controls, differentiated 3T3-L1 transfectants over-expressing *Notch1* or *Notch4* showed lower levels of lactate released to the extracellular medium, whereas the differentiated transfectants over-expressing *Notch3* released higher levels of lactate (Fig. 4F). No significant differences were observed in cells over-expressing *Notch2*, *Dlk1* or *Dlk2*, or in non-transfected 3T3-L1 differentiated cells (Fig. 4D,E,F).

**Figure 5:**
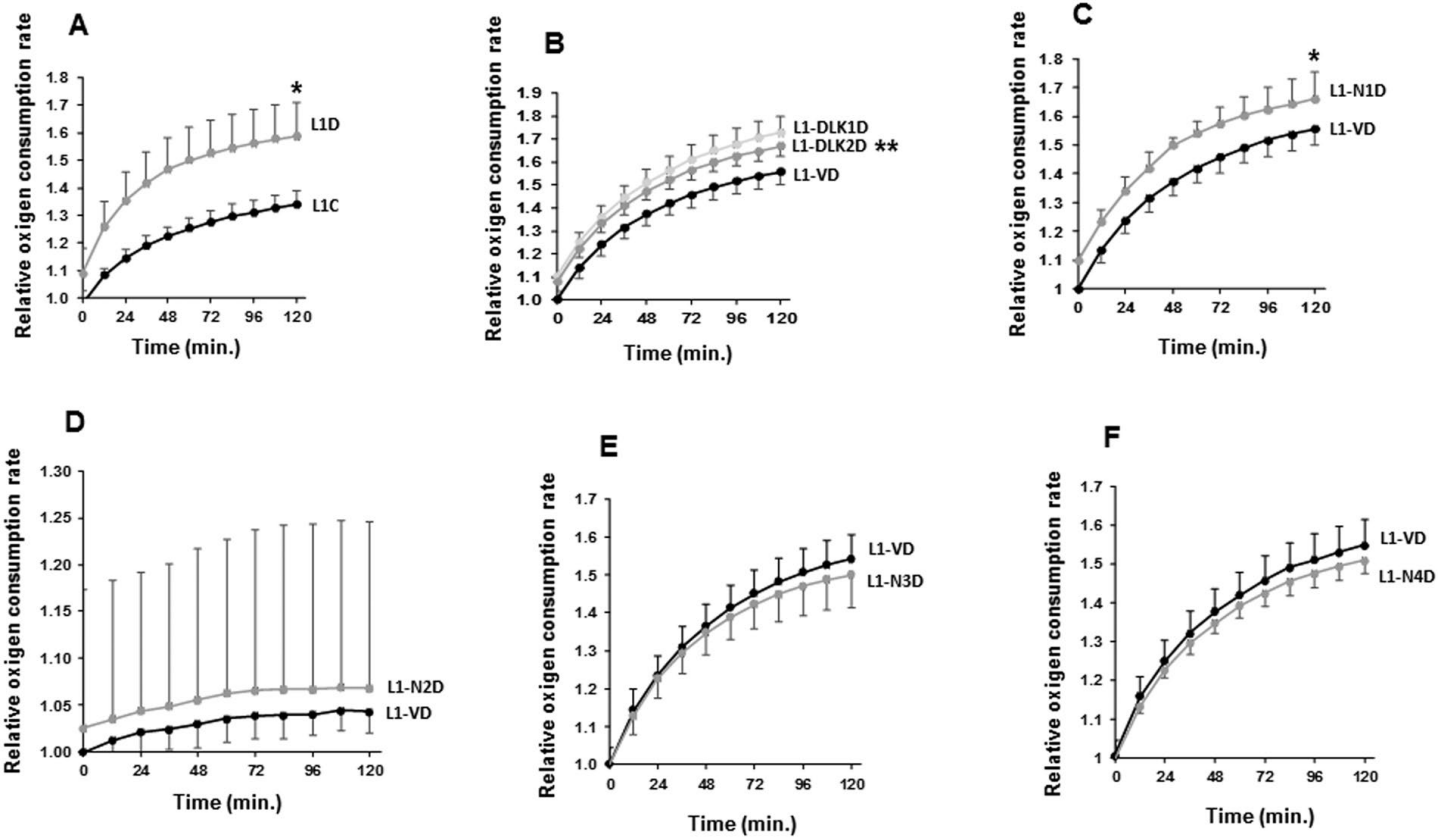
Oxygen consumption rate (OCR) in 3T3-L1 adipocytes over-expressing *Dlk* and *Notch* genes. Analysis of the relative oxygen consumption rate (OCR) in non-transfected 3T3-L1 cells (**A**) and 3T3-L1 cells over-expressing *Dlk1* or *Dlk2* genes (**B**), and *Notch1* (**C**), *Notch2* (**D**), *Notch3* (**E**) or *Notch4* genes (**F**). The fold activation or inhibition was calculated relative to the time 0 of seven-day differentiated non-transfected or empty-vector-transfected cells, which was set arbitrarily at 1. Data are shown as the mean ± SD of at least three biological assays performed in triplicate. The statistical significance calculated by Student’s t-tests is indicated at 120 minutes (*p ≤ 0.05, **p ≤ 0.01, ***p ≤ 0.001).

Finally, we measured the rate of oxygen consumption (OCR), which is a measure of cellular respiration and mitochondrial function, by using a phosphorescent oxygen probe (Fig. 5). As shown, differentiation of 3T3-L1 cells into adipocytes increased the oxygen consumption rate (OCR) (Fig. 5A). Besides, we observed higher levels of OCR in *Dlk1*, *Dlk2*, or *Notch1* transfectants when compared with their respective controls (Fig. 5B,C). On the other hand, the OCR change in 3T3-L1 transfectants over-expressing *Notch2*, *Notch3* and *Notch4* was not significant (Fig. 5D,E,F).

### The proteins DLK1 and DLK2 inhibit the activation and signaling of each one of the four mammalian NOTCH receptors

Since both DLK proteins inhibit adipogenesis of 3T3-L1 cells (Supplementary Figure 2) and inhibit NOTCH1 activation and signaling^36^, we considered important to study here whether DLK1 or DLK2 could block the observed proadipogenic effects of the over-expression of NOTCH1 in 3T3-L1 cells. For this purpose, we performed standard adipogenic assays with 3T3-L1 cells stably over-expressing the NOTCH1 receptor, cultured with control conditioned media or conditioned media containing sDLK1 or sDLK2 proteins during the entire adipogenic treatment. Soluble DLK proteins in the media were able to diminish the increment in the adipogenic potential of 3T3-L1 cells generated by the over-expression of *Notch1*, as indicated by a decrease in *aP2* and *Ppar*g expression (Supplementary Figure 3B). These results indicate that soluble DLK proteins can inhibit the 3T3-L1 adipogenesis process through the inhibition of NOTCH1 signaling.

**Figure 6:**
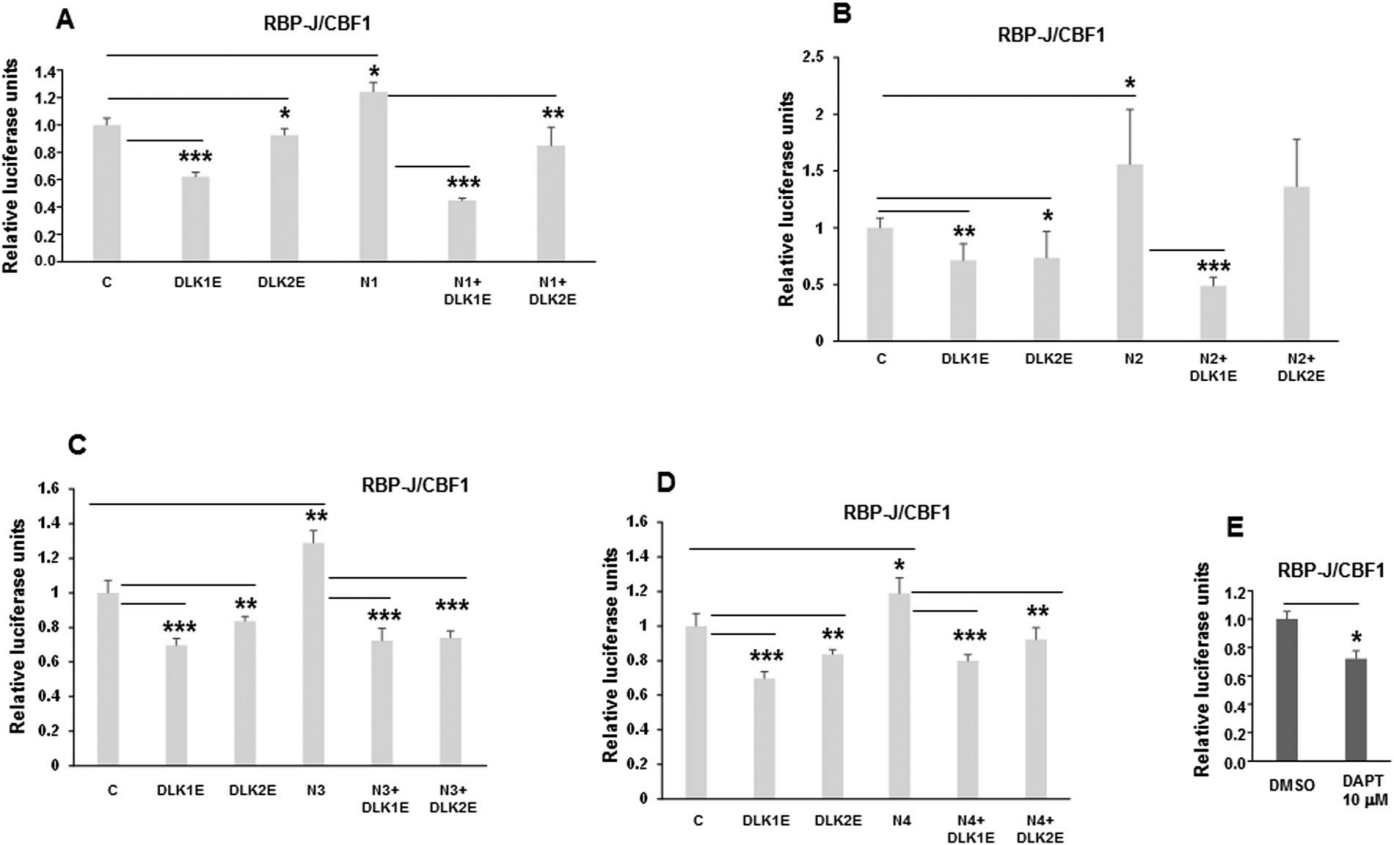
The proteins DLK1 and DLK2 inhibit the activation of each one of the four NOTCH receptors. NOTCH transcriptional activity, as measured by gene reporter luciferase assays, was determined in Balb/c14 cells transiently co-transfected with a plasmid driving the expression of each NOTCH receptor: N1 (**A**), N2 (**B**), N3 (**C**) and N4 (**D**); and/or a plasmid driving the expression of DLK1 or DLK2 extracellular regions (**A**–**D**). (**E**) Analysis of NOTCH transcriptional activity in Balb/c14 cells treated with DMSO or with the inhibitor DAPT (10 micromolar). The fold-activation or inhibition in these luciferase assays is measured relative to the control (**C**), set arbitrarily at 1. Data are shown as the mean ± SD of at least three biological assays performed in triplicate. The statistical significance of Student’s t-tests results is indicated (*p ≤ 0.05, **p ≤ 0.01, ***p ≤ 0.001).

Following this observation, we decided to study whether DLK proteins could also affect the activation of the other three NOTCH receptors. We analyzed NOTCH-dependent transcriptional activity by luciferase assays in Balb/c14 cells transiently expressing the extracellular regions of DLK1 (DLK1E) or DLK2 (DLK2E), alone or in combination with one of the four full length NOTCH receptor genes (Fig. 6). The results indicated that transient over-expression of DLK1 or DLK2 extracellular regions inhibited NOTCH1, 3 and 4 signaling to different extents (Fig. 6A,C,D). DLK1 also seemed to strongly inhibit NOTCH2 signaling, whereas no significant differences were found in NOTCH2 signaling when DLK2 was transiently over-expressed (Fig. 6B). We could observe that the grade of inhibition caused by DAPT, a gamma-secretase complex inhibitor, was similar to that observed when each DLK protein was over-expressed (Fig. 6E). These results indicate that DLK proteins are able to inhibit the activity of the four mammalian NOTCH receptors to different extents, and suggest that the effects of NOTCH receptors on adipogenesis and adipocyte browning can be modulated by membrane and soluble DLK proteins.

### Feedback modulation of gene expression among NOTCH receptors and DLK proteins

**Figure 7:**
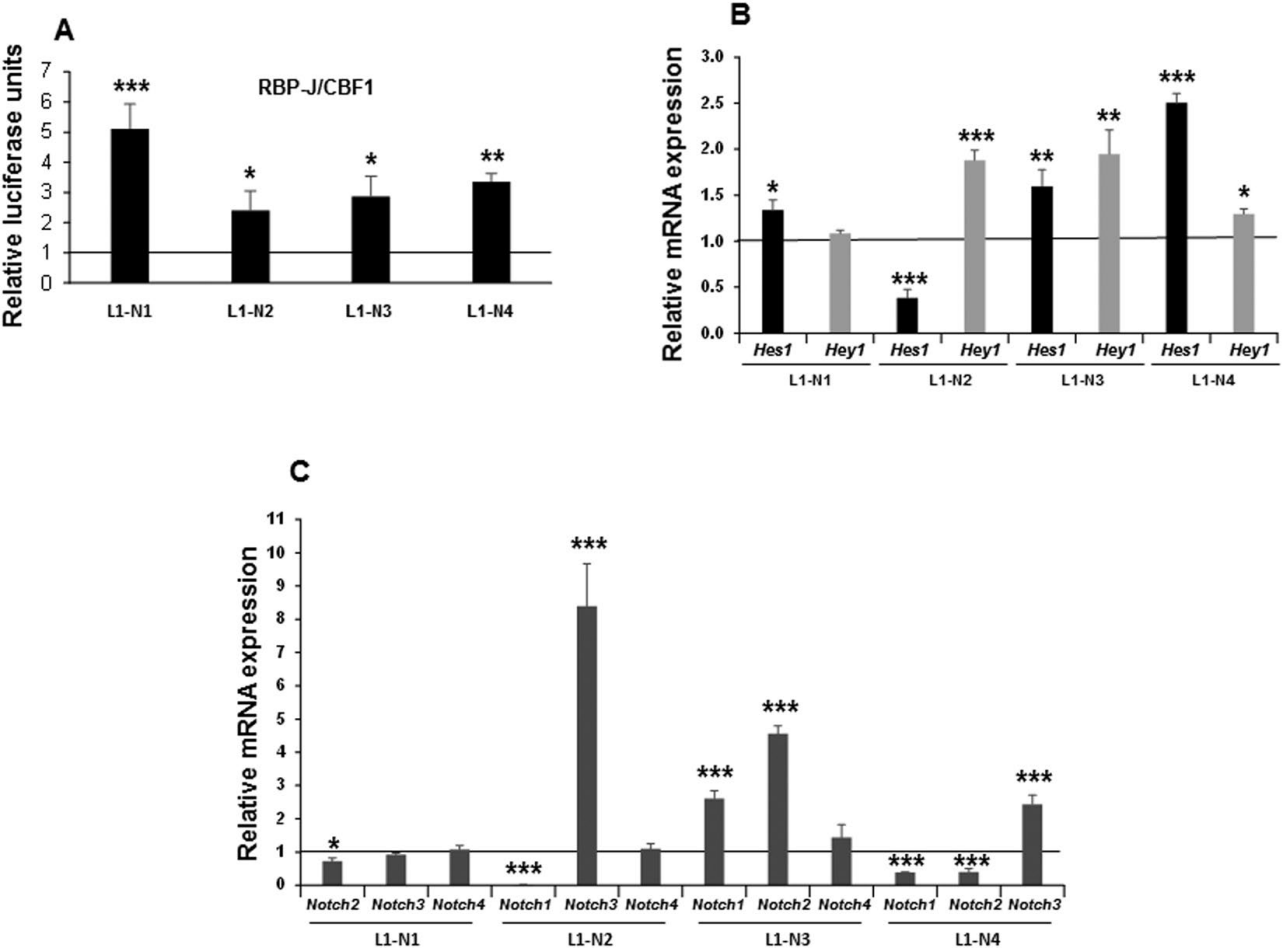
NOTCH activation and signaling in 3T3-L1 cells stably over-expressing each one of the four NOTCH receptors. (**A**) qRT-PCR analysis of the relative *Hes1* and *Hey1* mRNA expression levels in the stable *Notch1* gene transfectant (L1-N1), the stable *Notch2* gene transfectant (L1-N2), the stable *Notch3* gene transfectant (L1-N3), and the stable *Notch4* gene transfectant (L1-N4). (**B**) NOTCH transcriptional activity, as measured by gene reporter luciferase assays, in these four *Notch* genes stable transfectants. (**C**) qRT-PCR analysis of the relative individual *Notch* mRNA expression levels in stable *Notch1* gene transfectant (L1-N1), the stable *Notch2* gene transfectant (L1-N2), the stable *Notch3* gene transfectant (L1-N3), and the stable *Notch4* gene transfectant (L1-N4). The relative luciferase activities were always normalized with renilla values and referred to those of control cells. Data in all qRT-PCR assays were normalized to *P0* mRNA expression levels. The fold activation or inhibition in all assays is measured relative to the empty vector control, set arbitrarily at 1. Data are shown as the mean ± SD of at least three biological assays performed in triplicate. The statistical significance of Student’s t-tests results is indicated (*p ≤ 0.05, **p ≤ 0.01, ***p ≤ 0.001).

**Figure 8:**
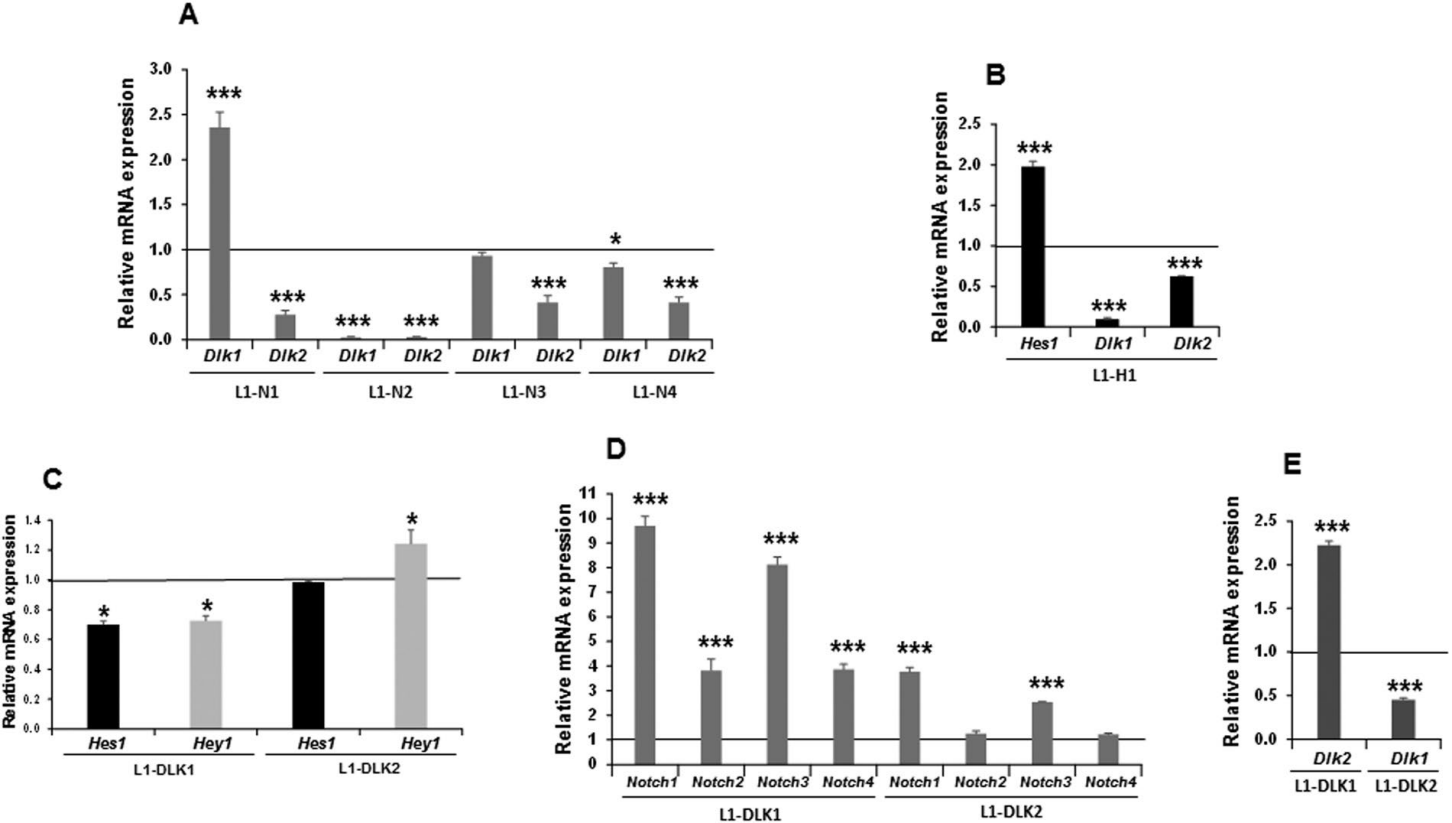
Feedback modulation among *Notch* and *Dlk* gene expression in 3T3-L1 preadipocytes. (**A**) qRT-PCR analysis of the relative individual *Dlk* (**B**) mRNA expression levels in the stable *Notch1* gene transfectant (L1-N1), the stable *Notch2* gene transfectant (L1-N2), the stable *Notch3* gene transfectant (L1-N3), and the stable *Notch4* gene transfectant (L1-N4). (**B**) qRT-PCR analysis of the relative *Hes1* and *Dlk* mRNA expression levels in the stable *Hes1* gene transfectant (L1-H1). (**C**) qRT-PCR analysis of the relative *Hes1* and *Hey1* mRNA expression levels in the stable *Dlk1* gene transfectant (L1-DLK1) and the stable *Dlk2* gene transfectant (L1-DLK2). qRT-PCR analysis of the relative *Notch* (**D**) and *Dlk* (**E**) mRNA expression levels in the stable *Dlk1* gene transfectant (L1-DLK1), and the stable *Dlk2* gene transfectant (L1-DLK2). In all qRT-PCR assays, data were normalized to *P0* mRNA expression levels. The fold activation or inhibition in all assays was measured relative to the empty vector, set arbitrarily at 1. Data are shown as the mean ± SD of at least three biological assays performed in triplicate. The statistical significance of Student’s t-tests results is indicated (*p ≤ 0.05, **p ≤ 0.01, ***p ≤ 0.001).

The inhibitory effect of DLK proteins on all NOTCH receptors’ activity, and additional published data confirming the inhibition of *Dlk1* expression by NOTCH signaling^4,5^, suggested to us the existence of a complex feedback mechanism that would modulate the overall expression levels of DLK proteins and NOTCH receptors, as well as NOTCH activation and signaling. This feedback modulation could influence the adipogenic potential and the final adipocyte phenotype of 3T3-L1 cells. To analyze this potential feedback mechanism, we used our 3T3-L1 transfectant pools over-expressing each one of the four NOTCH receptors, the NOTCH target gene *Hes1* or each one of the two DLK proteins (Figs 7 and 8).

We first observed that the over-expression of any of the four NOTCH receptors resulted in an increase in NOTCH-dependent transcriptional activity (Fig. 7A), which indicated that all NOTCH receptors were transcriptionally active in our cells. As expected, stable over-expression of NOTCH1, NOTCH3 and NOTCH4 in 3T3-L1 cells increased the expression of the NOTCH signaling target genes *Hes1* and *Hey1*. However, NOTCH2 over-expression increased *Hey1*, but surprisingly inhibited *Hes1* expression (Fig. 7B). This fact suggested to us that changes in *Notch2* expression levels might influence the expression of the other three *Notch* genes, which, in turn, may affect the global NOTCH signaling and, ultimately, *Hes1* expression. We show in Fig. 7C that over-expression of *Notch1* exerted no significant effect on the expression of *Notch3* and *Notch4*, but caused a small but significant decrease in *Notch2* expression. *Notch2* over-expression caused no significant changes in *Notch4* mRNA levels, but dramatically increased the expression of *Notch3* and decreased that of *Notch1*. On the other hand, over-expression of *Notch3* up-regulated the expression of *Notch1* and *Notch2*, but did not cause significant changes in *Notch4* expression. Finally, *Notch4* over-expression increased the expression of *Notch3*, but down-regulated that of *Notch1* and *Notch2*. Thus, a variation in the expression of only one of the *Notch* genes seems to modulate the expression of the others in a way that could allow cells to reach a particular stoichiometry in the levels of global NOTCH activity.

We also studied here whether the alteration of *Notch* expression could affect the expression of *Dlk1* and *Dlk2*. We observed that over-expression of each *Notch* gene decreased *Dlk2* expression in different amounts (Fig. 8A). *Notch2* and *Notch4* also decreased *Dlk1* expression, but no significant changes were observed in *Dlk1* expression levels following *Notch3* over-expression. Surprisingly, *Notch1* over-expression in these cells was accompanied by a significant increase in *Dlk1* levels (Fig. 6A). NOTCH target genes, such as *Hes1*, whose expression has previously been inversely correlated with *Dlk1* gene expression levels^5^, could mediate some of the observed effects of every NOTCH receptor signaling on *Dlk1* and *Dlk2* gene expression. Thus, we have observed that *Hes1* over-expression in 3T3-L1 cells strongly decreased the expression of *Dlk1*, as published previously, and, to a lower extent, that of *Dlk2* (Fig. 8B).

We show here that the over-expression of *Dlk1* led to a significant decrease in *Hes1* and *Hey1* expression. However, despite DLK2 also inhibiting NOTCH1, 3 and 4 receptors’ transcriptional activity, no significant changes in *Hes1* expression and even a slight increase in *Hey1* expression were observed when *Dlk2* was over-expressed in these cells (Fig. 8C). The slight effects of *Dlk2* over-expression on *Hes1* and *Hey1* expression suggested to us that the response of 3T3-L1 cells to the inhibition of NOTCH signaling by DLK proteins could lead also to a change in the expression pattern of *Notch1–4*. Indeed, *Dlk1* and *Dlk2* over-expression led to variations in each *Notch* gene expression to different extents (Fig. 8D). Finally, we observed here that the over-expression of one *Dlk* gene affects the expression of the other (Fig. 8E), as described before^21,36^.

All these data suggest that the expression of *Notch* and *Dlk* genes seems to be coordinated in the cells by a mechanism that globally regulates the NOTCH transcriptional activity of each NOTCH receptor and signaling to permit or not a particular biological process, such as white or brown adipogenesis, to proceed.

## Discussion

The role of NOTCH signaling in adipogenesis is still unclear. Different authors have claimed either null, positive, or negative effects on the adipogenesis of different cell lines, among them 3T3-L1 preadipocytes. Thus, some works suggested that the NOTCH signaling pathway is dispensable for adipocyte specification and differentiation from either mesenchymal or epithelial progenitors^1^. However, Garcés and coworkers showed that *Notch1* expression and function were necessary for adipogenesis of 3T3-L1 cells^2^. A more recent work described that the intracellular active region of NOTCH4 also enhanced 3T3-L1 cell proliferation and adipogenesis, and decreased *Dlk1* expression^4^. On the contrary, blocking NOTCH signalling with DAPT, an inhibitor of the gamma-secretase complex, enhances adipogenesis of differentiated mASCs at an early stage^46^. This effect may be due to depression of *Dlk1*/*Pref-1* and promotion of PPAR-gamma activation, which works through the inhibition of NOTCH2-HES1 pathway by DAPT. Other authors demonstrated that the expression of the gamma-secretase complex, which activates NOTCH signaling, decreases during adipocyte differentiation, and ectopic expression of bovine PSENEN protein reduced the adipogenesis process in 3T3-L1 cells^47^. Urs and collaborators proposed an initial requirement of NOTCH signaling inactivation for preadipocyte cell commitment^6^. The adipocyte differentiation of 3T3-L1 cells was significantly reduced when cells constitutively expressed JAGGED1 or HES1^5^. Finally, recent work indicate that increased NOTCH signaling in mouse adipocytes results in the blockage of the expansion of white adipose tissue^48^. We hypothesize that all these contradictory results could be attributed to the need of the cells to reach a precise level of each NOTCH receptor activation leading to a stoichiometry of global NOTCH signaling permissive or not for adipogenesis.

In this work, we studied the role of the NOTCH receptors and their non-canonical ligands, DLK1 and DLK2, in 3T3-L1 cells adipogenesis and adipocyte browning. We selected 3T3-L1 cells for these studies because they are one of the main cellular models of adipogenesis in which other authors have observed a high variability in the effects of NOTCH receptors and ligands. Our results show that stable over-expression of any of the four NOTCH receptors in 3T3-L1 preadipocytes enhances the adipogenic response of these cells. The lipolysis assays performed indicated that over-expression of the four NOTCH proteins increases the release of glycerol into the culture medium in response to isoproterenol, compared with their respective controls. Moreover, adipocytes showed different phenotypes depending on the *Notch* gene over-expressed. Thus, the over-expression of *Notch2*, *Notch3* or *Notch4* generated adipocytes with larger lipid droplets than controls, but *Notch1*-overexpressing adipocytes contained smaler multilocular lipid droplets as compared with its control cells. Thus, *Notch1* overexpression, but not *Notch* 2–4 overexpression, seems to induce a brown-like adipocyte phenotype in 3T3-L1 cells.

An increasing number of works by us and others have confirmed that DLK1 causes the inhibition of NOTCH signaling in different cellular processes^7,35,38–43^. In previous works, we have demonstrated that DLK1 and DLK2 can inhibit the adipogenic process of 3T3-L1 cells^17,49^, and that one of the molecular partners that could mediate the function of DLK proteins in adipogenesis was the NOTCH1 receptor, whose activation and signaling was inhibited by both DLK proteins in a dose-dependent manner^21,36,39,43^. We hypothesized here that DLK proteins could inhibit not only the activation and signaling of NOTCH1, but that of the four mammalian NOTCH receptors to different extents, thus generating a global level of NOTCH signaling permissive or not of the adipogenesis process. In agreement with this hypothesis, in this work we have demonstrated that soluble DLK1 and DLK2 proteins, as it is also the case with the membrane variants, reduce the augmented adipogenic potential of 3T3-L1 cells that over-express the NOTCH1 receptor. These results are in agreement with recent work from other authors demonstrating that blockage of the NOTCH1 receptor by DLK1 modulates the size of adipocytes *in vivo*^7^. As expected, we found that the over-expression of *Dlk1* in these cells decreases the amount of glycerol released from adipocytes into the culture medium in response to isoproterenol, which indicates that these adipocytes foster a lower lipolytic activity despite showing larger lipid droplets than control cells. Alternately, these adipocytes may have accumulated lower lipid levels throughout their adipogenic process. However, *Dlk2*-over expressing adipocytes also show large lipid droplets, although the release levels of glycerol were not significantly different. Moreover, we have also demonstrated that the DLK1 and DLK2 proteins inhibit the activity of each one of the four mammalian NOTCH receptors to different degrees, with the exception of NOTCH2, which was only inhibited by DLK1 but not by DLK2.

Even though 3T3-L1 preadipocytes have always been considered a well-known cellular model of white adipogenesis, Morrison and McGee observed that 3T3-L1 adipocytes display phenotypic characteristics of multiple adipocyte lineages, express *Pgc1a* and increase oxygen consumption and the expression of brown adipocyte genes in response to catecholamines^44^. In agreement with these authors, we have observed that the expression of several brown adipogenic markers is increased in 3T3-L1 adipocytes and the size and distribution of their lipid droplets is more coherent with that of brown or beige adipocytes. Despite a decreased expression of *Cidea* and *Sirt1*, differentiated 3T3-L1 cells show increased levels of *Ucp1*, *Pgc1a*, *Gyk* and *Prdm16* markers. These results, together with the multilocular small lipid droplets observed in these cells, are more consistent with a brown-like phenotype, as other authors have described^50^. The *Pparg* transcription factor is considered both as an adipocyte marker in 3T3-L1 cells and as a factor that favors adipocyte browning by activating *Ucp* expression^51^. Therefore, the induction of *Pparg* expression after 3T3-L1 differentiation also supports the idea that 3T3-L1 adipocytes show a brown-like phenotype. As expected, the level of glycerol release and the respiration rate were generally higher in differentiated adipocytes than in undifferentiated 3T3-L1 cells; however, not significant differences were observed for lactate output in 3T3-L1 adipocytes^44,52^.

The brown adipose tissue (BAT) functions by generating heat through mitochondrial uncoupling proteins, in particular UCP1 and UCP3^53^. The role of NOTCH signaling on brown adipogenesis is also controversial. Thus, some authors showed that inhibition of NOTCH signaling promotes browning and alleviates obesity^8,9^. On the contrary, Pasut and co-workers demonstrated that, subsequent to the deletion of the transcription factor PAX7 (Paired Box 7) and following acute muscle injury, NOTCH signaling promoted the differentiation of satellite cells into brown adipocytes rather than into a skeletal muscle cell phenotype^10^. Some works have also shown a role for DLK1 in adipocyte browning and suggest a possible involvement of DLK2 in this process. Thus, the expression of *Dlk1* is high in fetal BAT and declines after birth, although *Dlk1*-null mice showed unaltered BAT fetal development, with an over-activation of thermogenesis in the postnatal period. As it occurs in 3T3-L1 preadipocytes, *Dlk1* expression decreases after the induction of the differentiation in brown preadipocyte cell lines^33^. Other authors described that *Dlk1* inhibits thermogenesis in brown adipocyte models^34^. Young women with cold-activated brown adipose tissue have lower serum DLK1 than women lacking brown adipose tissue^54^.

It is believed that an increase in the expression of brown-fat signature genes and in the number of mitochondria or in mitochondrial biogenesis and function are key features of BAT. Interestingly, we have observed that *Notch1* over-expression in differentiated 3T3-L1 cells upregulates the expression of *Ucp1*, *Pgc1a*, *Gyk*, *Prdm16*, *Cidea*, *Pparg* and *Sirt1*, and there is more amplification of *CytB* than in control cells, whereas their expression or amplification is down-regulated or it does not change when *Notch2* or *Notch*3 are over-expressed in these cells. In the case of *Notch4* transfectants, the expression of *Ucp1*, *Pgc1a*, *Gyk*, *and Pparg* markers is down-regulated in differentiated cells, and the amplification of *CytB* is also lower than control. However, unexpectedely, the expression of *Prdm16*, *Cidea* and *Sirt1* is increased in *Notch4* transfectants. CIDEA is a multifunctional protein, highly expressed in brown adipose tisusue, with a clearly defined role in the promotion of lipid droplets in brown and white adipocytes^50^. These data suggest that *Notch1* may promote differentiation into 3T3-L1 brown adipocytes, whereas *Notch2*, *Notch3* and *Notch4* may promote 3T3-L1 cells to differentiate toward the white phenotype. It has been described that brown adipocytes showed higher levels of OCR and ECAR when compared with control cells^55^. We observed that only *Notch1* adipocytes showed significantly higher levels of OCR and diminished levels of lactate released into the culture medium. Our findings may imply that lactate could be transported to mitochondria and used as a fuel substrate. Interestingly, a recent study described that lactate was a major substrate for the TCA cycle in several tissues, including adipocytes and glucose feeds the TCA cycle via circulating lactate^56^. Besides,the increased number of mitochondria in *Notch1*-over-expressing cells would lead to a greater use of lactate as fuel in these cells. This suggests that only *Notch1*-overexpressing adipocytes could undergo functional lineage transformation toward a brown adipocyte phenotype. All these data suggest that *Notch1*-over-expressing transfectants could have developed smaller lipid droplets than their empty vector-transfected control adipocytes because of a higher expression of UCP1 and increased mitochondrial biogenesis, which would lead to a higher OCR and lipolysis rate.

On the other hand, we have observed that differentiated 3T3-L1 cells over-expressing *Dlk1* show decreased levels of *Cidea* and *Sirt1* and increased expression levels of *Pgc1a* and *Gyk*, although no significant changes in *Ucp1* and *Prdm16* expression were observed. The expression of *Gyk*, *Cidea* and *Sirt1* decreases in stable *Dlk2-*over-expressing transfectants, but no significant changes were observed in *Pgc1a*, *Ucp1* or *Prdm16* expression. Therefore, these transfected cells, when differentiated, do not show evidence of a gene expression profile typical of brown adipose cells, despite the increase in *Pgc1a* and *Gyk* expression in *Dlk1*-stably transfected cells. Besides, the levels of mitochondrial *CytB* in *Dlk2* transfectants, but not in *Dlk1* transfectants, are lower than in control cells, although, as it occurs with *Notch*1 transfectants, both *Dlk1* and *Dlk2* transfectants show higher OCR than control cells. It has been described that DLK1-overexpressing mice showed increased oxygen consumption, indicating an increase in energy expenditure^29^. These slight and contradictory effects could be probably explained by a differential inhibition of each NOTCH receptor´activity, since each NOTCH receptor exerts different effects on the expression of these markers. We think that qualitative and quantitative differences in overall NOTCH signaling may place the cells in different states allowing them to interpret the same extracellular signals so that they differentiate or not to at least two different adipocyte phenotypes.

Finally, in this work we have also revealed the existence of a complex feedback mechanism that modulates the overall expression levels of the *Notch* and *Dlk* genes and the global NOTCH activation and signaling. Each NOTCH receptor increases the level of global NOTCH signaling and induces the expression of the NOTCH signaling targets *Hes1* and *Hey1*, except for *Notch2*, which unexpectedly reduces *Hes1* mRNA levels. Furthermore, stable over-expression of each one of the NOTCH receptors in 3T3-L1 cells influences the expression levels of the other *Notch* genes to different extents. This interplay among *Notch* members, specifically between *Notch1* and *Notch3*, has been shown by other authors^57,58^. We have also shown here that DLK proteins inhibit each one of the four NOTCH receptors’ activity to different extents and that the over-expression of *Dlk1* inhibits the global NOTCH signaling by reducing the level of expression of *Hes1* and *Hey1*. The observed unexpected effects of stable *Dlk2* over-expression on *Hes1* and *Hey1* suggest that the potential inhibitory effect of the stable over-expression of *Dlk2* on NOTCH signaling could be achieved through other factors different from HES1 and HEY1.

NOTCH signaling can both positively and negatively regulate canonical ligand expression, such that defects in NOTCH signaling are associated with increased expression of DLL1^59^ or DLL4^60^. As other authors have indicated^5^, non-canonical ligands such as DLK1 are also regulated by NOTCH signaling. In this work we have observed that NOTCH receptors’ activity modulate *Dlk1* and *Dlk2* expression levels to different extents. Besides, the results presented here demonstrate that *Hes1* over-expression in 3T3-L1 cells inhibits the expression of both *Dlk2* and *Dlk1*. These data are in agreement with those of Ross and coworkers, who demonstrated that HES1 seemed to down-regulate *Dlk1* expression, which would permit adipogenesis of 3T3-L1 cells to complete^5^.

Importantly, it has been reported that the expression of *Dlk1* and *Dlk2* is coordinated in 3T3-L1 cells and that DLK1 and DLK2 proteins interact and may inhibit each other’s activities^21,36,39,43^. Indeed, in this work, we have observed that the over-expression of one *Dlk* gene affects to the expression of the other. Therefore, it is also possible that, depending upon the different expression levels of DLK1 and DLK2 in these cells, both proteins may lead either to a decrease or to an increase in the global NOTCH signaling levels, depending on their stoichiometry and their interaction affinities. This kind of coordination of the expression and competition between NOTCH ligands has been also described by other authors^61^. Furthermore, we have observed that the over-expression of *Dlk1* and *Dlk2* increases the expression of endogenous *Notch* genes to different extents, suggesting a response of the cells aimed at re-equilibrating global NOTCH signaling. Our data reveal that the interplay among the NOTCH receptors and DLK proteins in 3T3-L1 cells affect the expression of each one of these genes, and suggest that the expression of the NOTCH canonical and non-canonical ligands participates in these complex feed-back mechanisms. Further analysis, beyond the scope of this work, will be needed to study in detail the coordination in the expression of these genes in 3T3-L1 preadipocytes.

**Figure 9:**
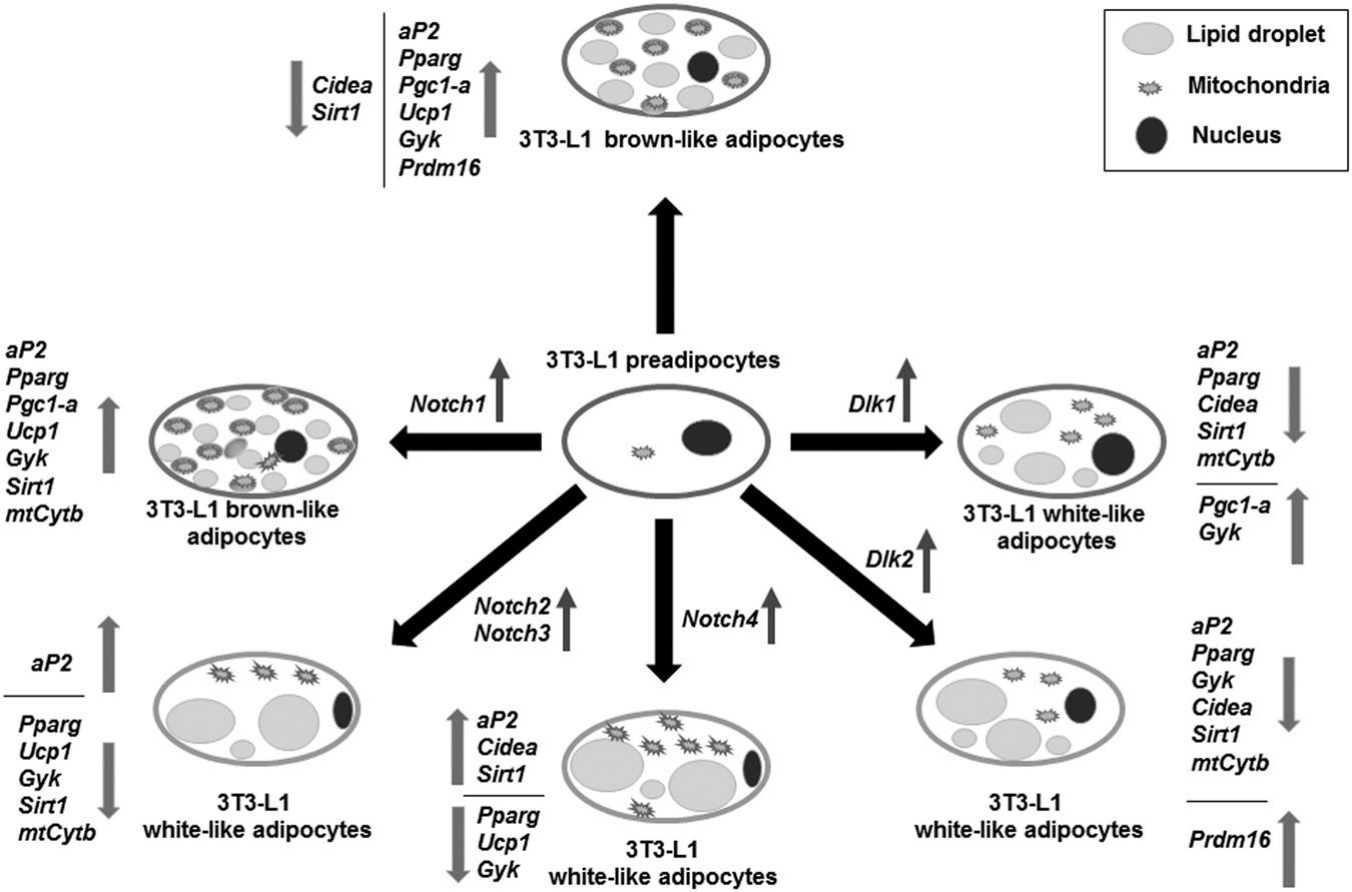
Outline of the role of *Notch* and *Dlk* gene over-expression on 3T3-L1 adipogenesis and adipocyte browning. The effects of adipogenic inductors on control 3T3-L1 preadipocytes is compared with the effects of the same adipogenic inductors on 3T3-L1 cells stably over-expressing each one of the *Notch* or *Dlk* genes. The direction (up or down) of the variations in the expression of *aP2*, *Pparg*, and several brown adipocyte and mitochondrial biogenesis markers, are shown by arrows.

In summary, the data presented here deepen into the understanding of the role of NOTCH and DLK proteins on the control of 3T3-L1 adipogenesis and adipocyte fate. In Fig. 9, we schematically summarize the potential effects of NOTCH receptors and DLK proteins on 3T3-L1 adipogenesis and preadipocyte whitening/browning. We believe that continuing with the study of the molecular pathways involved in this cell differentiation process and the coordination among the four NOTCH signaling activities and their modulation by canonical and non-canonical ligands would permit to advance in the development of novel and promising anti-obesity therapies.

## Methods

### Plasmids

Bacterial cultures, plasmid DNA isolation and purification, and bacterial transformation and amplification of TOP10 *Escherichia coli* competent cells was performed as previously described^36^. Plasmids pCDLK1 (DLK1) and pCDLK2 (DLK2) derivate from pCD2 vector (V1) and contain the complete cDNA sequence of either *Dlk1* or *Dlk2* in sense orientation, respectively^17^. Plasmid pC-N1 (N1) derivates from pCD2 (V1) and contains the complete mouse *Notch1* cDNA (ATCC clone: MBA-105) in sense orientation^36^. Plasmid pCN-N2 (N2) (A gift from Dr. Anna Bigas, IMIM, Barcelona, Spain) contains the complete mouse *Notch2* cDNA cloned into the *Eco*RI-*Not*I restriction sites of the pCDNA3 expression vector (V2). Plasmid pEntry-N3 (N3) contains the complete mouse *Notch3* cDNA sequence in pCMV6-Entry vector (Origene) (V3). pG-N4 (N4) contains the complete mouse *Notch4* cDNA sequence cloned into the *Hind*III-*Xba*I restriction sites of the pEGFP-N1 vector (Clontech) (V4). Plasmid pN-HES1 (H1) expresses the complete HES1 protein^36^. Plasmids pL-DLK1 (DLK1E) and pL-DLK2 (DLK2E) contain de cDNA of the extracellular regions of DLK1 and DLK2^36^. All plasmids were confirmed by restriction analysis and sequenced by Macrogen. The oligonucleotides used for sequencing were obtained from Bonsai Technologies.

### Cell culture and cell transfections

Mammalian cell lines were cultured as describe before^17,36^. The cell lines used were: 3T3-L1 (L1; ATCC CCL-92.1); HEK 293 T/17 (ATCC CRL-11268); and Balb/c14 (Balb/c 3T3 sub-clones negative for *Dlk1* expression^62^. Stable and transient transfections were performed as previously described^36^.

### Quantitative RT-PCR expression analysis

Confluent cell monolayers were processed as previously described to obtain total RNA and cDNAs^36^. Gene expression assays by qRT-PCR were performed as previously described^36^. *P0* expression was used as the control to compare the C_T_ from the different samples in all qPCR experiments^63^. The qRT-PCR primers to determine *Dlk1*, *Dlk2* and *Hes1* expression levels were previously described^17,21^. The expression of *Notch* genes and *Hey1* gene was analyzed with the oligonucleotides showed in Table 1 (Bonsai Technologies).

**Table 1:** Oligonucleotides used in qRT-PCR assays for the expression of the *four Notch* genes and the *Hey1* gene.

*Notch1* E: 1,068	mNotch1-up: 5′-GCTGAGCATGTACCCGAGC-3′
	mNotch1-down: 5′-ATCACGCTTGAAGACCACGTT-3′
*Notch2* E: 1,0464	mNotch2-up: 5′-TTGGGCAGGTTACATCCAGTTCCT-3′
	mNotch2-down: 5′-AGCCAGGACCATGCCAAACATTTC-3′
*Notch3* E: 0.9796	mNotch3-up: 5′-TGCAGTCAGCTGAGAATGACCACT-3′
	mNotch3-down: 5′-ACATCCCGAAGTGGGTATGGGAAA-3′
*Notch4* E: 1,0174	mNotch4-up: 5′-AAGGCCAAAATAACCGTTAAGCT-3′
	mNotch4-down: 5′-ACCGGACATCCTAAACCCTCTT-3′
*Hey1*	mHey1-up: 5′-ATGTGGCCTACTTCAGCTCCATGT-3′
	mHey1-down: 5′-TCTCCAGGCAGGTAAACAATGGGA-3′

Oligonucleotide amplification efficiencies of *Notch* primers (E) are shown.

### Conditioned media

HEK 293T/17 cells were transiently transfected with plasmids pL-DLK1 (DLK1E) and pL-DLK2 (DLK2E), which express the soluble forms of DLK1 (sDLK1) and DLK2 (sDLK2), respectively. Protein expression and secretion of these proteins were analyzed as previously described^36^ (Supplementary Figure 3A).

### Protein sample preparation and Western blotting

Protein samples were obtained, quantified and electrophoresed as previously described^36^. Western blot was performed by using an appropriated dilution of the primary and the secondary antibodies (usually, 1: 2,000) (Table 2). Detection of alpha-tubulin with a specific antibody (Sigma) was used as a protein loading control.

**Table 2:** Primary and secondary antibodies used in Western blot analysis.

Protein	Dilution of primary and secondary antibodies	Company
NOTCH1	Rabbit anti-NOTCH1 C20R (1:1000)	Santa Cruz Biotechnology
Active NICD1	Rabbit anti-Cleaved NOTCH1 (Val 1744) (1:000)	Cell signaling
NOTCH2	Goat anti-NOTCH2 M20 (1:500)	Santa Cruz Biotechnology
NOTCH3	Rabbit anti-NOTCH3 (1:1000)	Abcam
NOTCH4	Rabbit anti-NOTCH4 (1:1000)	Upstate Millipore
HA	Mouse anti-HA 16B12 (1:5000)	Covance
DLK1	Rabbit anti-DLK1 (1:1000)	Nueda *et al*.^49^
DLK2	Rabbit anti-DLK2 (1:500)	Proteintech
α-tubulin	Mouse anti-alpha-Tubulin (1:5000)	Sigma

### Luciferase assays

NOTCH transcriptional activity was analyzed by luciferase assays in Balb/c14 cells and stably transfected 3T3-L1 pools as previously described^36^. When cells were transiently co-transfected with DLK expression plasmids, the assays were performed in Balb/c14 cells because these cells do not express DLK1 and express very low levels of DLK2, and have higher transient transfection efficiency compared to 3T3-L1 cells. To analyze the effect of DLK proteins on NOTCH activity, we performed these assays by co-transfecting Balb/c14 cells with a *Notch* expression plasmid and with plasmids expressing sDLK1 or sDLK2 proteins. We also treated Balb/c14 cells with the gamma-secretase inhibitor DAPT (10 μM) as a NOTCH signaling inhibition control. Transfected cells were processed as previously described^36^.

### Adipogenic assays

The induction of 3T3-L1 adipogenesis and the staining of adipocytes with red oil O was performed according to standard procedures as previously described^17^. Sometimes, cells were induced to differentiate in the presence of control conditioned media or conditioned media containing sDLK1 or sDLK2 during the entire differentiation process. Assays were repeated at least three times.

We determined the level of differentiation by analyzing the expression of the late adipocyte differentiation marker *aP2* and the intermediate marker *Pparg*, as previously described^64^, seven days after induction of adipogenesis. To study the phenotype of 3T3-L1 adipocytes, we also analyzed in the same samples the expression of the following brown adipose markers: *Ucp1* (coding for uncoupling protein-1); *Pgc1a (*Peroxisome proliferator-activated receptor-gamma coactivator); *Gyk*, encoding for a glycerol kinase activated in brown adipocytes and involved in triglyceride and glycerophospholipid synthesis^65^; *Prdm16*, thought to function as a key transcriptional co-regulator of brown cell adipogenesis function^55^; *Cidea*, highly expressed in lipid droplet membranes and mitocondria of brown adipocytes,involved in the browning process and considered as a BAT differentiation marker^45^; and *Sirt1*, a mitochondrial biogenesis marker^66^. Pre-designed qRT-PCR oligonucleotides of these genes were obtained from Sigma (Table 3).

**Table 3:** Oligonucleotides used in qRT-PCR assays to quantify the expression of the genes indicated.

Gene	Oligonucleotide sequences
*Ucp1*	mUcp-1up: 5′-CAATTGTACAGAGCTGGTAAC-3′
	mUcp1-down: 5′-TGTTTTTACCACATCCACGT-3′
*Gyk*	mGyk-up: 5′-ATCTATGGCCTAATGAAAGC-3´
	mGyk-down: 5′-GAAAATACACACTTATGGCCC-3′
*Pgc1a*	mPgc1-a-up: 5′-TCCTCTTCAAGATCCTGTTAC-3′
	mPgc1-a-down: 5′-CACATACAAGGGAGAATTGC-3′
*Prdm16*	mPrdm16-up: 5′-ATCTACAGGGTAGAAAAGCG-3′
	mPrdm16-down: 5′-TCTCCGTCATGGTTTCTATG-3′
*Cidea*	mCidea-up: 5′-GTGTTAAGGAATCTGCTGAG-3′
	mCidea-down: 5′-CTATAACAGAGAGCAGGGTC-3
*Sirt1*	mSirt1-up: 5′-AAACAGTGAGAAAATGCTGG-3′
	mSirt1-down: 5′-′GGTATTGATTACCCTCAAGC-3

We also analyzed the biogenesis of mitochondria by qPCR analysis of the ratio of the levels of the mitochondrial gene *CytB* and the genomic gene *ApoB* in terminal differentiated cells by using oligonucleotides previously described^67,68^.

The amount of glycerol released into the culture medium was determined using the lipolysis colorimetric assay kit (BioVision). The extracellular lactate was measured using the lactatate colorimetric assay kit (BioVision). Finally, to measure the oxygen consumtion rate (OCR), we used the oxigen consumption rate assay kit (AbCam). In these three last assays, we seeded 15,000 cells per well in 96-well plates, then we proceeded with the adipogenic differentiation protocol and, finally, we performed the assays following the manufacturer’s recommendations. Data were normalized with total protein amount or the number of cells seeded in each well.

### Equipment and Settings

RNA and DNA concentration and purity (20 nm/280 nm) were analyzed by using the spectrophotometer NanoDrop One (Thermo Scientific).

Quantitative RT-qPCRS were performed with StepOne Plus RT-qPCR system (Applied Biosystems) by using Fast SYBR green mix. The results were obtained by using the StepOne software 2.3 with the parameters recommended by the company, analyzing always the melting curve for each gene.

Microscopy images were visualized with an objective 40X of Motic AE31 microscopy connected to a Moticam 2300 camera (3.0 M Pixel USB 2.0). Cell images, 400X magnification, were acquired with the software Motic Images Plus 2.0 with the standard parameters (Exposition: 418.9, Gamma: 0.8019).

Western blot images s were obtained by developing exposed films (CP-BU New, Agfa) for 30 seconds (Tubulin), 1 minute (NOTCH1, NICD1, NOTCH2), 5 minutes (NOTCH3) or 10 minutes (NOTCH4), with the Pierce ECL Plus Western Blotting substrate kit (Thermo Scientific) in a Curix 60 developing apparatus (AGFA). Films were scanned with HP Officejet Pro 8600 scanner and signals of the different proteins were quantified by using the QuantityOne 4. 6. 5. (Basic) software. We analyzed the intensity of the signal per mm^2^ with a Volume Rect Tool. Blots for the Supplementary Figure 1 were converted to a grey color.

Colorimetric determinations to quantify total protein amount, extracellular lactate in the culture medium, and the release of glycerol to the the medium were performed with a plate reader Axis UVM340 (Biochrom). To measure the OCR of the different cultured adipocytes, we used a fluorescence spectrophotometer F-7000 (Hitachi).

Luciferase assays were measured by using a Monolight 3096 Microplate Luminometer (Becton Dickinson) and samples were processed with the Dual-224 Luciferase Reporter Assay System (Promega), following the supplier’s recommendations.

### Statistical analysis

Data are presented as the mean ± S.D of at least three different independent assays performed in triplicate. Data were also analyzed with Student’s *t* test to determine statistical significance. A *P* value of ≤0.05 was considered statistically significant (*); a *P* value ≤0.01 was considered highly statistically significant (**); and a *P* value of ≤0.001 was considered extremely statistically significant (***).

## Electronic supplementary material

Supplementary information
