# Author Correction: DLK proteins modulate NOTCH signaling to influence a brown or white 3T3-L1 adipocyte fate

**DOI:** 10.1038/s41598-018-36659-8

**Published:** 2018-12-07

**Authors:** María-Luisa Nueda, María-Julia González-Gómez, María-Milagros Rodríguez-Cano, Eva-María Monsalve, María José M. Díaz-Guerra, Beatriz Sánchez-Solana, Jorge Laborda, Victoriano Baladrón

**Affiliations:** 1 Área de Bioquímica y Biología Molecular, Dpto. Química Inorgánica, Orgánica y Bioquímica, Facultad de Medicina de Albacete/CRIB/Unidad de Biomedicina, Universidad de Castilla-La Mancha/CSIC, C/Almansa 14, 02008 Albacete, Spain; 2 Área de Bioquímica y Biología Molecular, Dpto. Química Inorgánica y Bioquímica, Facultad de Farmacia/CRIB/Unidad de Biomedicina, Universidad de Castilla-La Mancha/CSIC. C/Almansa 14, 02008 Albacete, Spain; 3 Laboratory of Cellular Oncology, National Cancer Institute (NCI), National Institutes of Health (NIH), Bethesda, MD USA

Correction to: *Scientific Reports* 10.1038/s41598-018-35252-3, published online 16 November 2018

The original version of this Article contained a typographical error in the spelling of the author María José M. Díaz-Guerra, which was incorrectly given as María-José M. Díaz-Guerra. This error has now been corrected in the PDF and HTML versions of this Article.

