# Supplementary material for: DLK proteins modulate NOTCH signaling to influence a brown or white 3T3-L1 adipocyte fate
Source: Sci Rep. 2018 Nov 16;8:16923. doi: 10.1038/s41598-018-35252-3 (PMC6240076; doi:10.1038/s41598-018-35252-3)

SUPPLEMENTARY INFORMATION

DLK proteins modulate NOTCH signaling to influence a brown or white 3T3-L1 adipocyte fate

María-Luisa NUEDA[2], María-Julia GONZÁLEZ-GÓMEZ[2], María-Milagros RODRÍGUEZ-CANO[2], Eva-María MONSALVE[1], María-José M. DÍAZ-GUERRA[1], Beatriz SÁNCHEZ-SOLANA[3], Jorge LABORDA[2] and Victoriano BALADRÓN[1#]

[1/2]**Permanent address**: Área de Bioquímica y Biología Molecular, Dpto. Química Inorgánica, Orgánica y Bioquímica, Facultad de Medicina de Albacete[1]/Facultad de Farmacia[2], CRIB/Unidad de Biomedicina, Universidad de Castilla-La Mancha/CSIC. C/Almansa 14, 02008 Albacete, Spain.

[3]**Present address**: Laboratory of Cellular Oncology, National Cancer Institute, National Institutes of Health, Bethesda, MD, EEUU.

[#]**Corresponding Author and Address**: Victoriano Baladrón, PhD. To whom correspondence should be addressed: E-mail Victoriano.Baladron@uclm.es. Telephone: +34967599200-Ext. 2926. Fax: +34967599327. Área de Bioquímica y Biología Molecular, Dpto. Química Inorgánica, Orgánica y Bioquímica, Facultad de Medicina de Albacete/CRIB/Unidad de Biomedicina, Universidad de Castilla-La Mancha/CSIC, C/Almansa 14, 02008 Albacete, Spain.

Supplementary figure 1. Stable over-expression of each one of the four NOTCH receptors in 3T3-L1 cells. (A) mRNA expression levels, represented by the 1/ΔCTxE (E: qRT-PCR amplification efficiency) of *Notch* genes in 3T3-L1 cells. qRT-PCR analysis of the relative mRNA expression levels (B, D, F and H) and densitometry analysis of the relative protein expression levels obtained by Western blot (C, G, E and I) in stable *Notch1* gene transfectant (L1-N1) (B and C), stable *Notch2* gene transfectant (L1-N2) (D and E), stable *Notch3* gene transfectant (L1-N3) (F and G), and stable *Notch4* gene transfectant (L1-N4) (H and I). In the case of *Notch1* gene transfectant we show the signal of non-active intracellular NOTCH1 and the signal of active intracellular NOTCH1 (NICD1) proteins. In the case of *Notch2* gene transfectant, we show the non-active intracellular NOTCH2 protein signal. In the case of *Notch3* gene transfectant, we show the complete NOTCH3 protein signal. In the case of *Notch4* gene transfectant, we show both the signal of complete and the non-active intracellular NOTCH4 proteins. The expression of alpha-tubulin was used as a loading control in all Western blots to normalize expression data. Representative Western blots are shown. Blot signals of the expression of the different proteins in the stable transfectants (Empty vector (L1-V1, 2, 3 or 4) and over-expressing transfectant) were cropped from original blots and delineated with horizontal white spaces (original blots for each protein signal are shown in Supplementary figure 4). Data in all qRT-PCR assays were previously normalized to *P0* mRNA expression levels. The fold activation in all assays is calculated relative to the empty-vector-transfected cells, set arbitrarily at 1. Data are shown as the mean ± SD of at least three biological replicates. The statistical significance of Student's t-tests results is indicated (* $p \leq 0.05$, ** $p \leq 0.01$, *** $p \leq 0.001$).

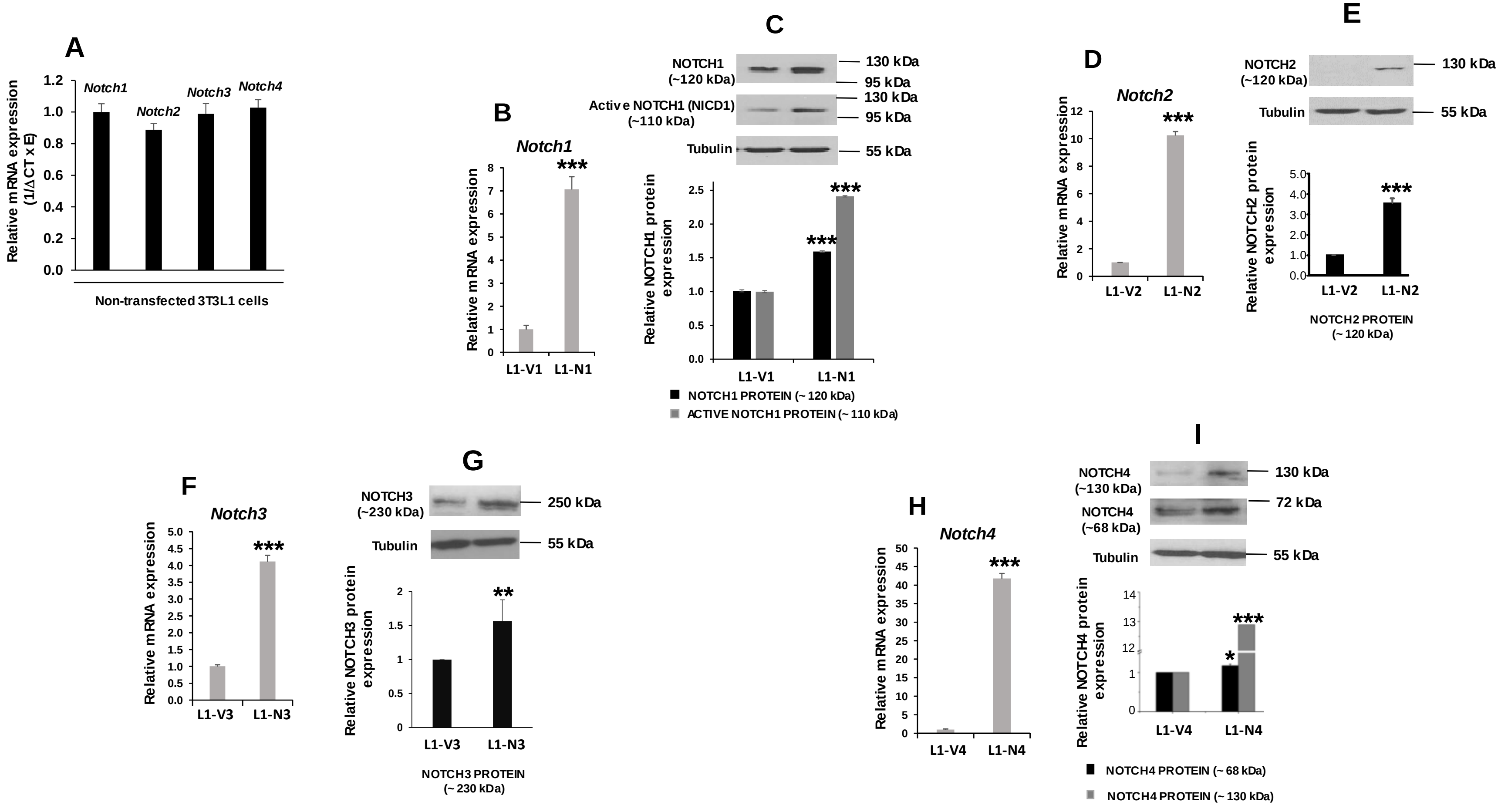
A
Relative mRNA expression (1/ΔCT x E)
Notch1
Notch2
Notch3
Notch4
Non-transfected 3T3L1 cells
B
Notch1
Relative mRNA expression
***
L1-V1
L1-N1
C
NOTCH1 (~120 kDa)
Active NOTCH1 (NICD1) (~110 kDa)
Tubulin
130 kDa
95 kDa
55 kDa
Relative NOTCH1 protein expression
NOTCH1 PROTEIN (~ 120 kDa)
ACTIVE NOTCH1 PROTEIN (~ 110 kDa)
D
Notch2
L1-V2
L1-N2
E
NOTCH2 (~120 kDa)
Relative NOTCH2 protein expression
NOTCH2 PROTEIN (~ 120 kDa)
F
Notch3
L1-V3
L1-N3
G
NOTCH3 (~230 kDa)
250 kDa
Relative NOTCH3 protein expression
**
NOTCH3 PROTEIN (~ 230 kDa)
H
Notch4
L1-V4
L1-N4
I
NOTCH4 (~130 kDa)
NOTCH4 (~68 kDa)
72 kDa
Relative NOTCH4 protein expression
*
NOTCH4 PROTEIN (~ 68 kDa)
NOTCH4 PROTEIN (~ 130 kDa)

Supplementary figure 2. Stable over-expression of DLK1 and DLK2 in 3T3-L1 cells. (A) mRNA expression, represented by the 1/ΔCTxE (E: qRT-PCR amplification efficiency) of *Dlk1* and *Dlk2* genes in 3T3-L1 cells. mRNA expression levels (B) and densitometry analysis of the relative protein expression levels obtained by Western blot (C), in stable *Dlk1* gene transfectant (L1-DLK1) and stable *Dlk2* gene transfectant (L1-DLK2). (D) qRT-PCR analysis of the relative mRNA expression levels of both *Dlk* genes in seven-day differentiated 3T3-L1 cells. (E) qRT-PCR analysis of the relative *aP2* and *Pparg* mRNA expression levels in seven-day differentiated stable *Dlk1* gene transfectant (L1-DLK1D) and stable *Dlk2* gene transfectant (L1-DLK2D). The expression of alpha-tubulin was used as a loading control in Western blots to normalize expression data. Representative Western blots are shown. Data in all qRT-PCR assays were previously normalized to *P0* mRNA expression levels. The fold activation in all assays is calculated relative to the empty vector transfected controls (L1-V1), set arbitrarily at 1. Data are shown as the mean ± SD of at least three replicates. The statistical significance of Student's t-tests results is indicated (* $p \leq 0.05$, ** $p \leq 0.01$, *** $p \leq 0.001$).

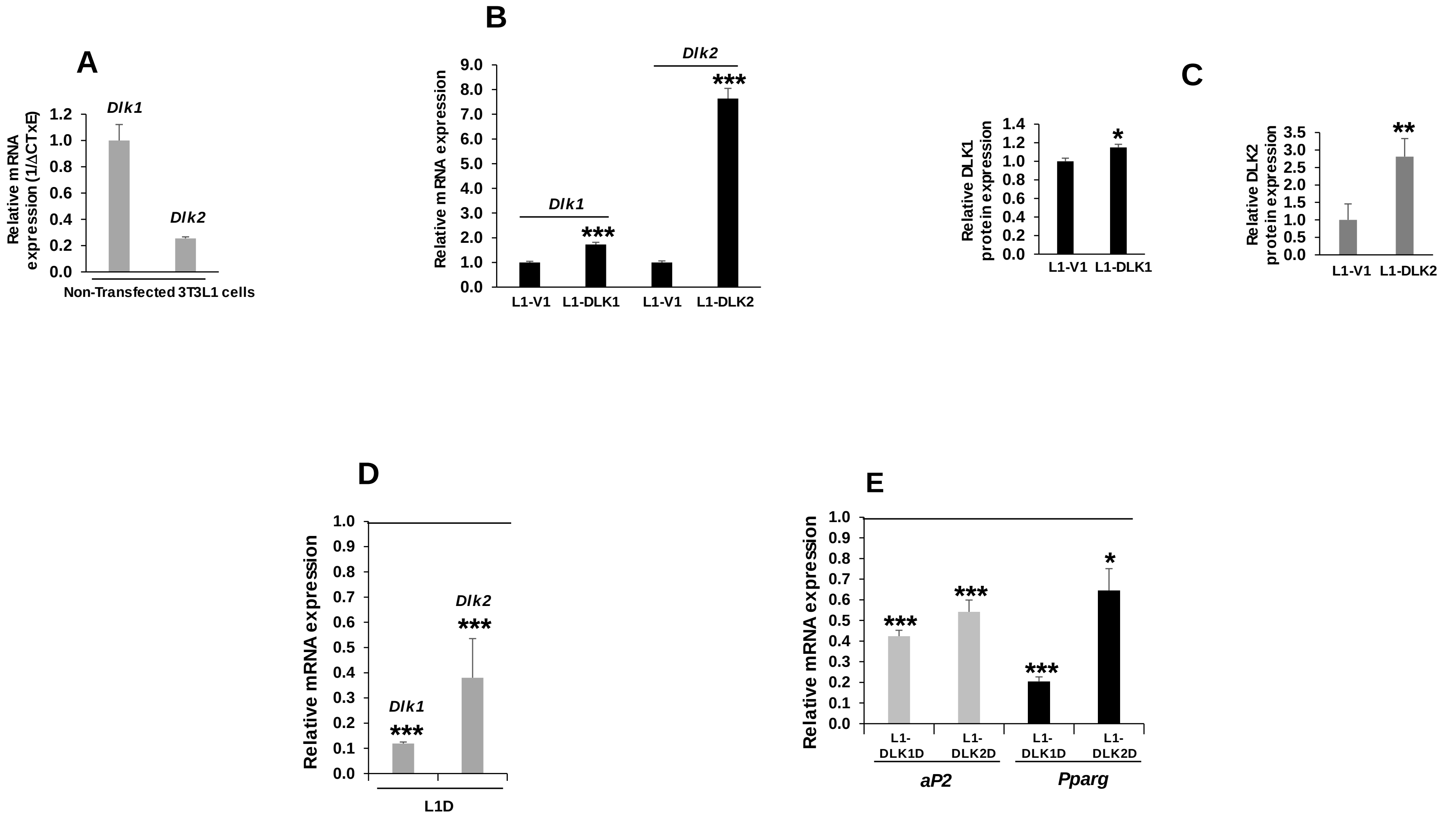
A
Dlk1
Dlk2
Relative mRNA expression (1/ΔCTxE)
1.2
1.0
0.8
0.6
0.4
0.2
0.0
Non-Transfected 3T3L1 cells
B
Dlk2
***
Dlk1
***
Relative mRNA expression
9.0
8.0
7.0
6.0
5.0
4.0
3.0
2.0
1.0
0.0
L1-V1
L1-DLK1
L1-V1
L1-DLK2
C
Relative DLK1 protein expression
1.4
1.2
1.0
0.8
0.6
0.4
0.2
0.0
*
L1-V1
L1-DLK1
Relative DLK2 protein expression
3.5
3.0
2.5
2.0
1.5
1.0
0.5
0.0
**
L1-V1
L1-DLK2
D
Relative mRNA expression
1.0
0.9
0.8
0.7
0.6
0.5
0.4
0.3
0.2
0.1
0.0
Dlk2
***
Dlk1
***
L1D
E
Relative mRNA expression
1.0
0.9
0.8
0.7
0.6
0.5
0.4
0.3
0.2
0.1
0.0
***
***
***
*
L1-DLK1D
L1-DLK2D
L1-DLK1D
L1-DLK2D
aP2
Pparg

Supplementary figure 3. Soluble DLK proteins inhibit adipogenesis of stably NOTCH1-over-expressing 3T3-L1 preadipocytes. (A) Representative Western blot analysis (complete blot showing these two soluble proteins) of culture media from HEK 293T-17 cells transfected with a construct expressing the extracellular soluble region of DLK1 or DLK2 proteins. sDLK1: conditioned medium (CM) containing soluble DLK1 protein (black arrow). sDLK2: conditioned medium containing soluble DLK2 protein (black arrow). CM Control: control conditioned medium. Analysis of the relative qRT-PCR expression of *aP2* and *Pparg* markers in seven-day differentiated 3T3-L1 cells stably over-expressing *Notch1* (L1-N1D) (B) in the presence of sDLK1, sDLK2, or control conditioned media. Data were previously normalized to *P0* mRNA expression levels. The fold activation or inhibition is calculated relative to the seven-day differentiated N1 cells (L1-N1D) incubated with control conditioned medium, set arbitrarily at 1. Data are shown as the mean ± SD of at least three replicates. The statistical significance of Student's t-tests results is indicated (* $p \leq 0.05$, ** $p \leq 0.01$, *** $p \leq 0.001$).

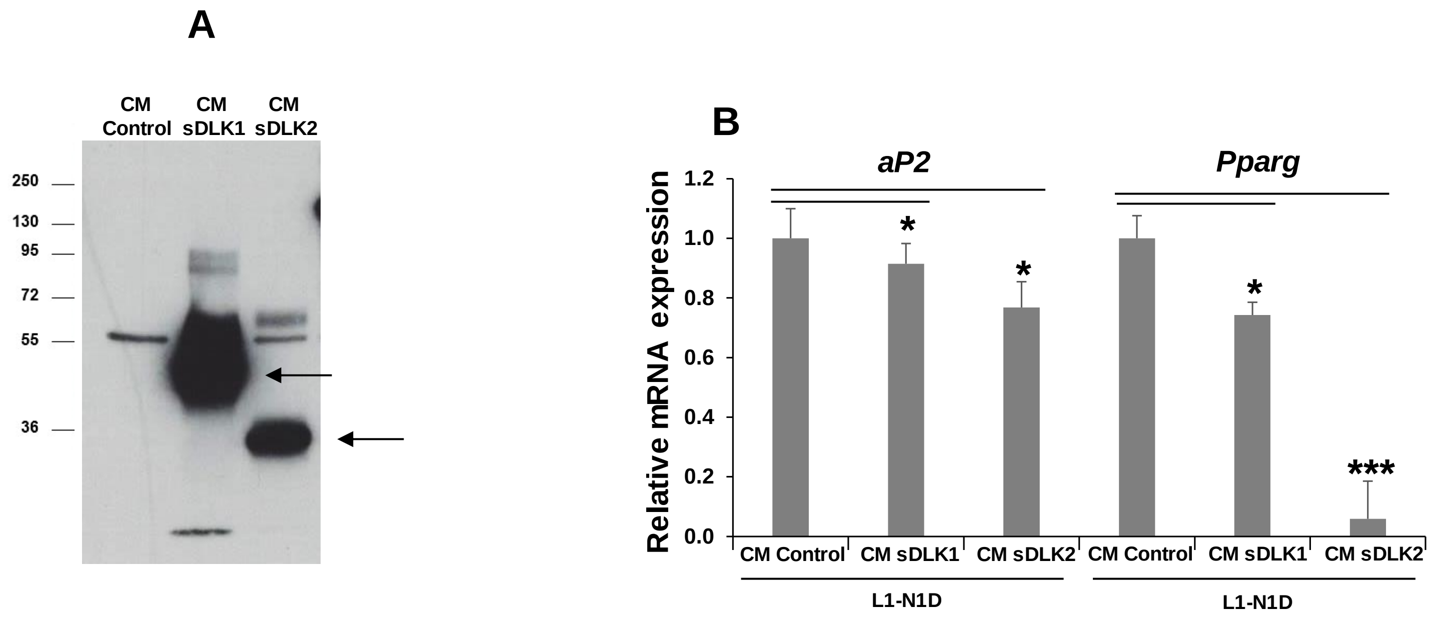
A
CM Control
CM sDLK1
CM sDLK2
250
130
95
72
55
36
B
aP2
Pparg
Relative mRNA expression
1.2
1.0
0.8
0.6
0.4
0.2
0.0
*
*
*
***
CM Control
CM sDLK1
CM sDLK2
CM Control
CM sDLK1
CM sDLK2
L1-N1D
L1-N1D

Supplementary figure 4. Original Western blots of NOTCH proteins and tubulin signals for the revised manuscript. In each film, we indicated the blot with the information for each NOTCH and tubulin protein. The arrows indicate the bands of the different proteins which were cropped to show in the Supplementary figure 1 and new Figures 1C and 1D. The antibodies used are shown in Table 2.

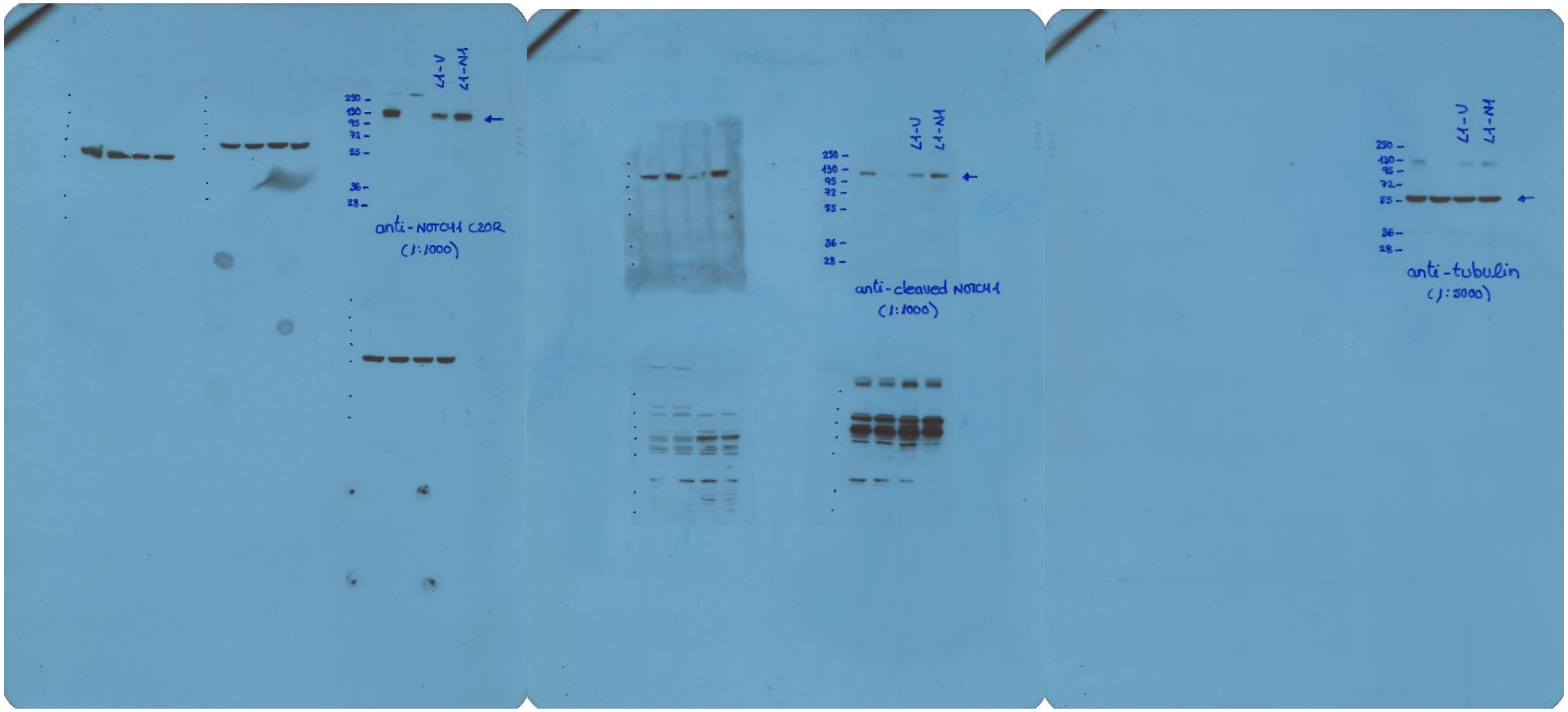

NOTCH1 blots showed in Supplementary figure 1

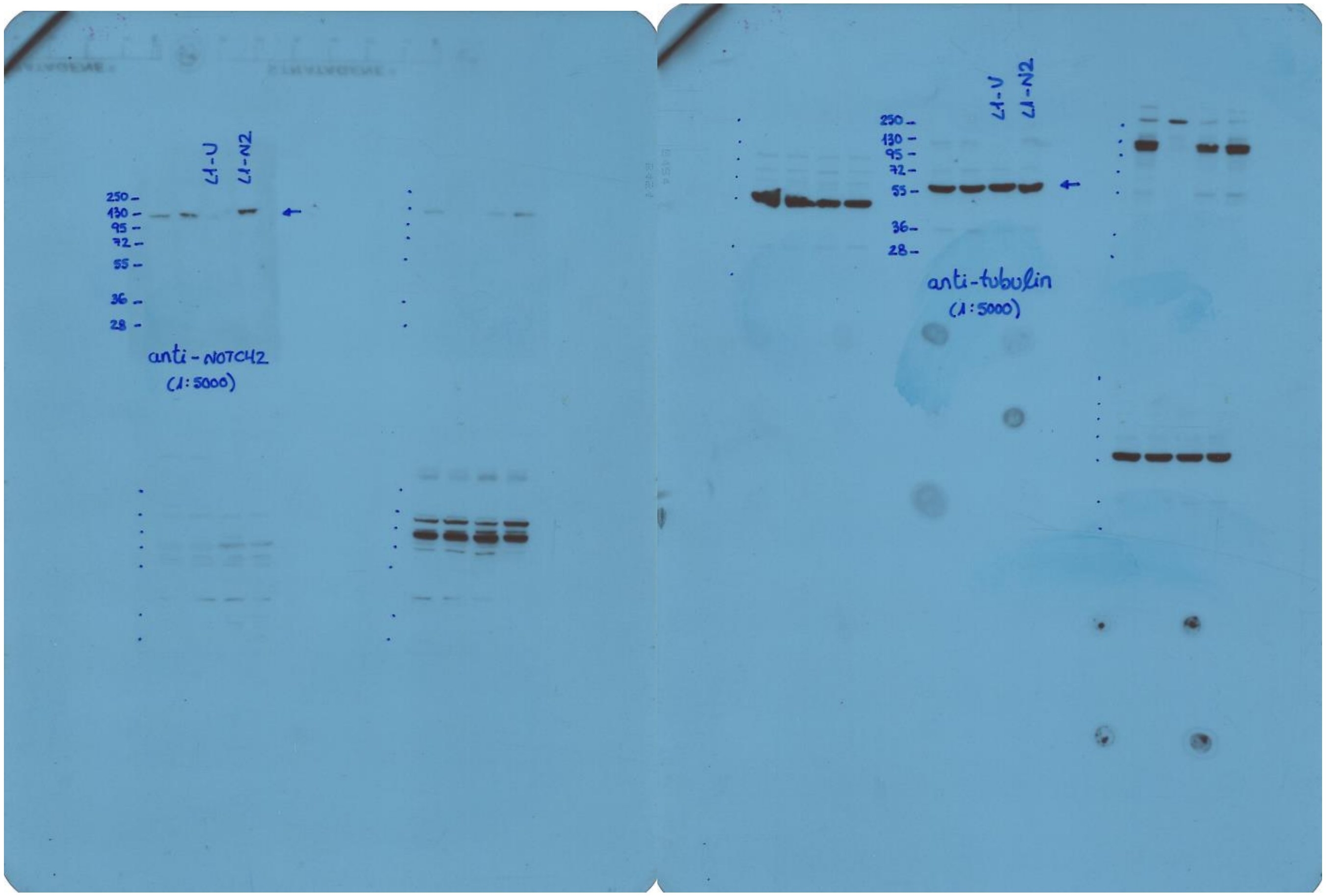

NOTCH2 blots showed in supplementary figure 1

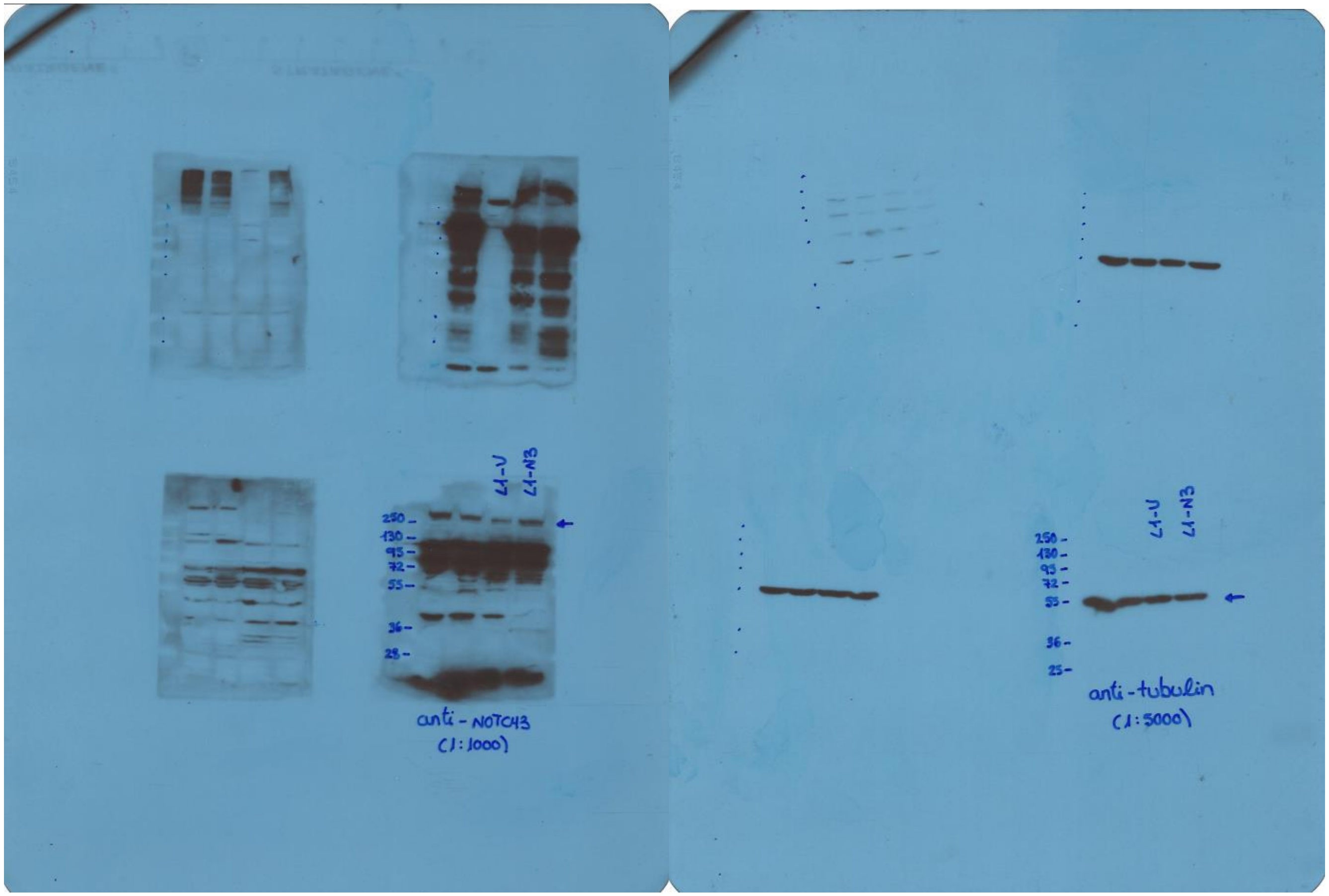

NOTCH3 blots showed in supplementary figure 1

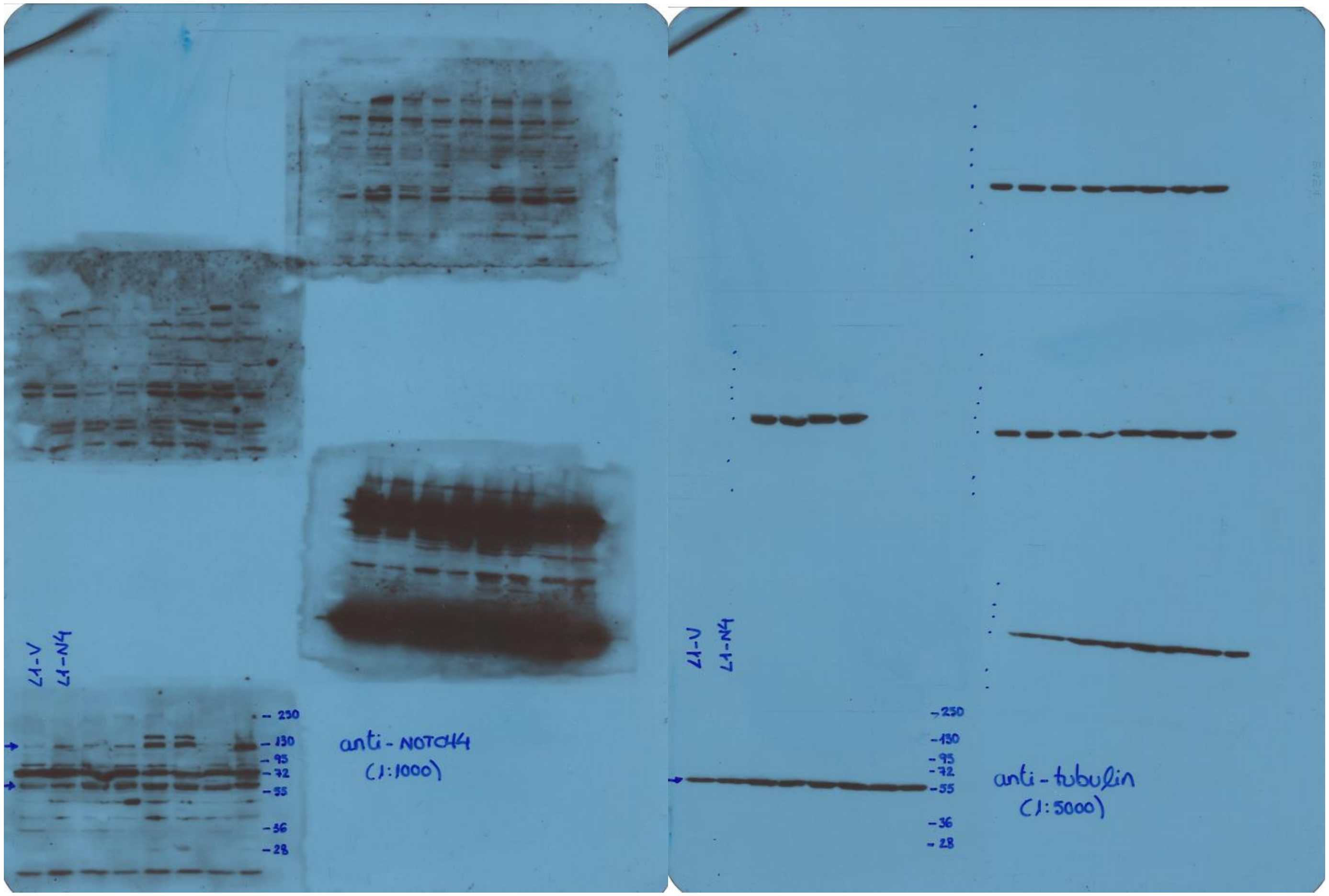

NOTCH4 blots showed in supplementary figure 1

NOTCH1 and NOTCH2 receptors and tubulin blots showed in figures 1C and 1D.

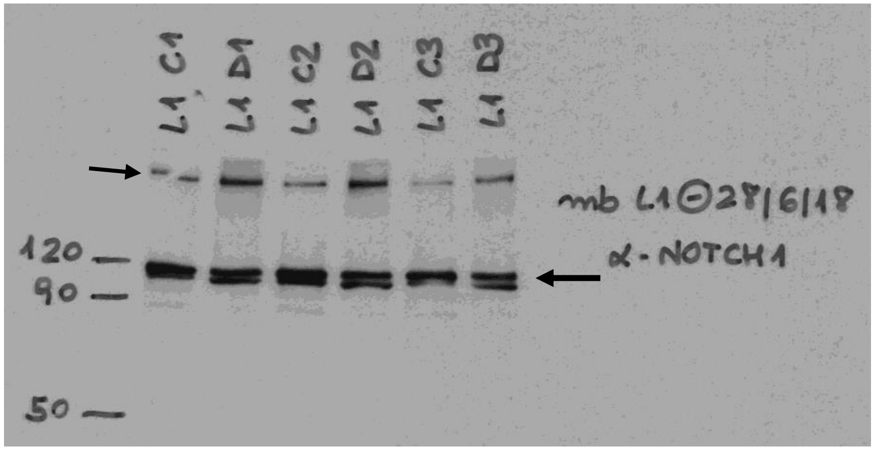

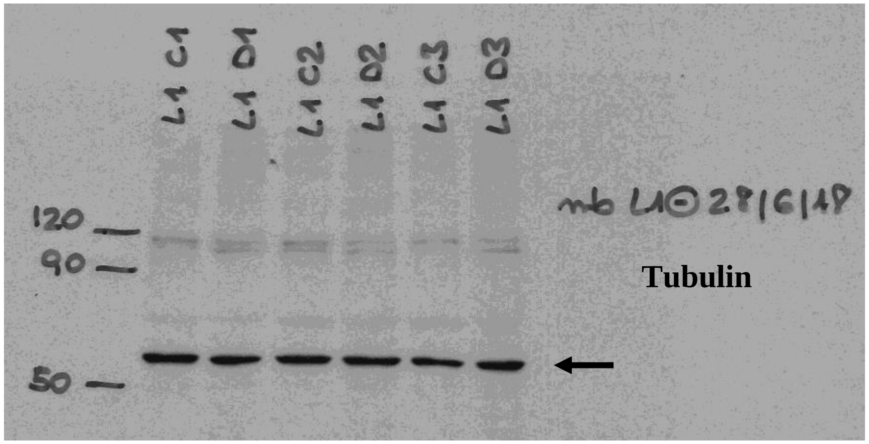

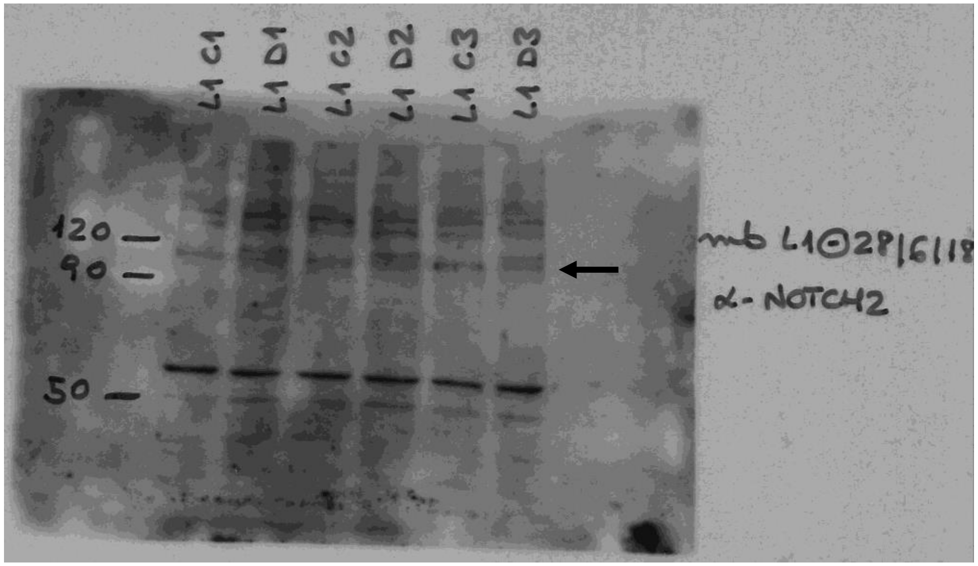

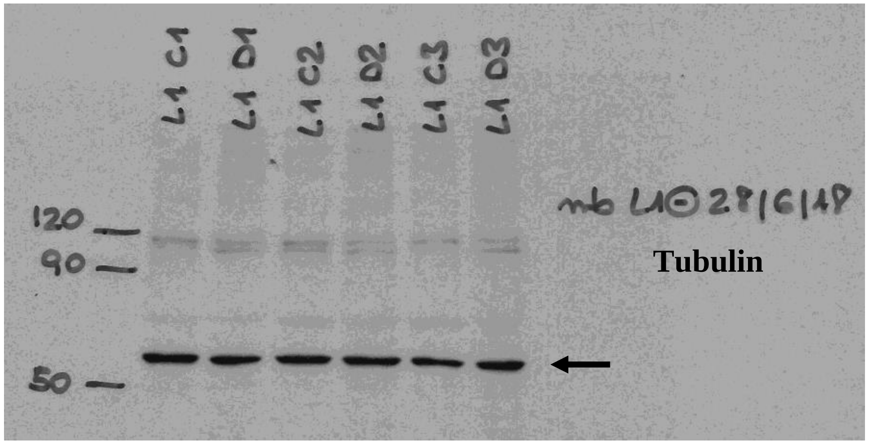

NOTCH3 and NOTCH4 receptors and their tubulin blots showed in figures 1C and 1D.

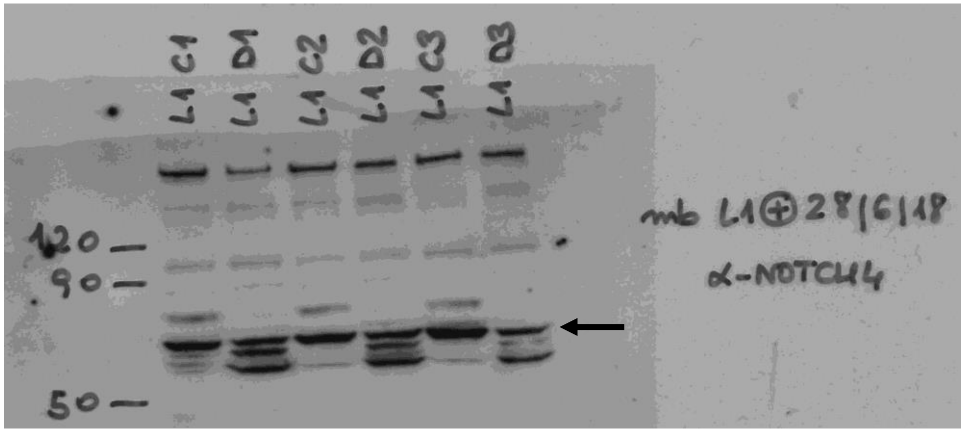

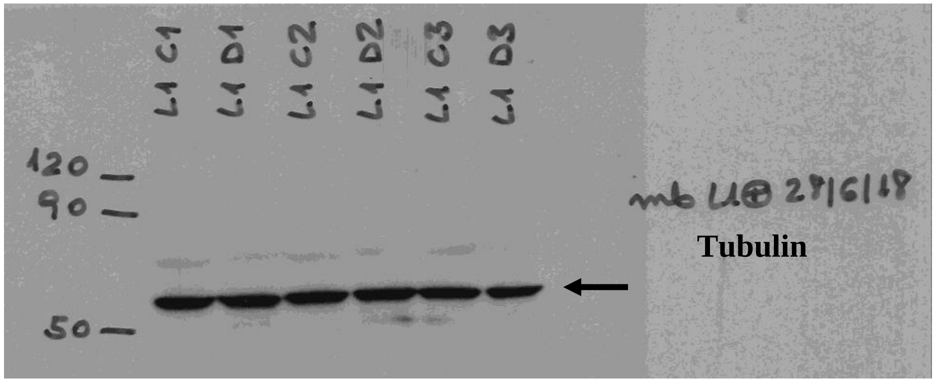

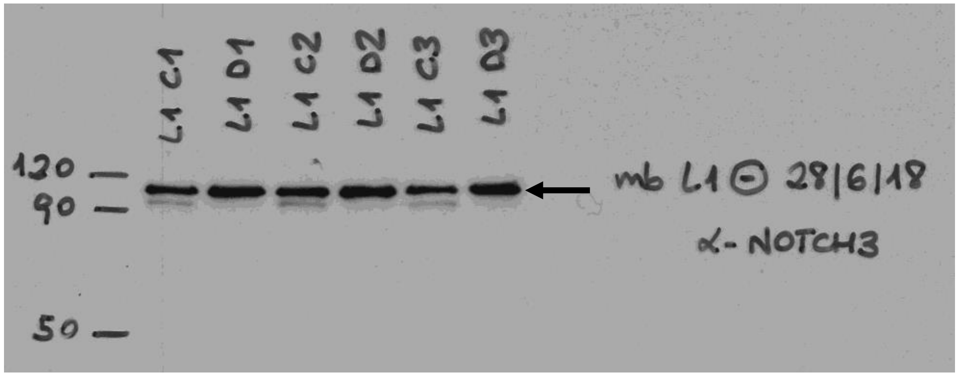

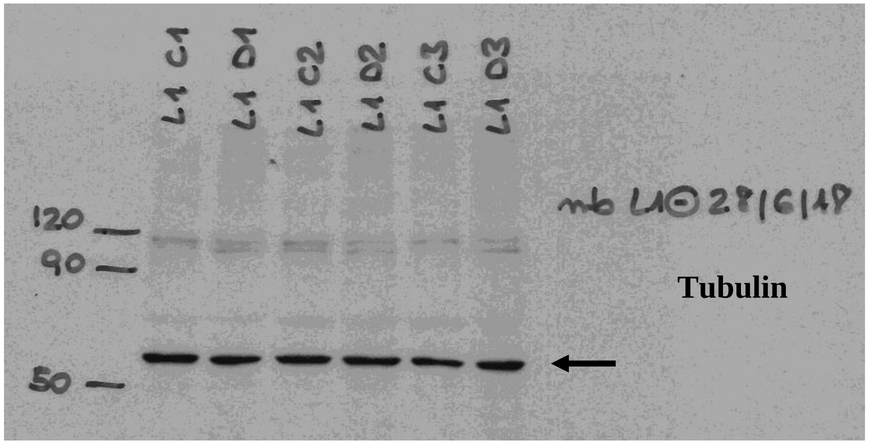

Supplement: Supplementary file 1 — Supplementary information [file 41598_2018_35252_MOESM1_ESM.pdf]
